# Isolation and Characterization of Lytic Bacteriophages Active against Clinical Strains of *E. coli* and Development of a Phage Antimicrobial Cocktail

**DOI:** 10.3390/v14112381

**Published:** 2022-10-28

**Authors:** Pavel Alexyuk, Andrey Bogoyavlenskiy, Madina Alexyuk, Kuralay Akanova, Yergali Moldakhanov, Vladimir Berezin

**Affiliations:** Laboratory of Antiviral Protection, Department of Virology, Research and Production Center for Microbiology and Virology, Almaty 050010, Kazakhstan

**Keywords:** *Escherichia coli*, bacteriophage, nosocomial infection, clinical strains, antibiotic resistance, phage cocktail

## Abstract

Pathogenic *E. coli* cause urinary tract, soft tissue and central nervous system infections, sepsis, etc. Lytic bacteriophages can be used to combat such infections. We investigated six lytic *E. coli* bacteriophages isolated from wastewater. Transmission electron microscopy and whole genome sequencing showed that the isolated bacteriophages are tailed phages of the *Caudoviricetes* class. One-step growth curves revealed that their latent period of reproduction is 20–30 min, and the average value of the burst size is 117–155. During co-cultivation with various *E. coli* strains, the phages completely suppressed bacterial host culture growth within the first 4 h at MOIs 10^−7^ to 10^−3^. The host range lysed by each bacteriophage varied from six to two bacterial strains out of nine used in the study. The cocktail formed from the isolated bacteriophages possessed the ability to completely suppress the growth of all the *E. coli* strains used in the study within 6 h and maintain its lytic activity for 8 months of storage. All the isolated bacteriophages may be useful in fighting pathogenic *E. coli* strains and in the development of phage cocktails with a long storage period and high efficiency in the treatment of bacterial infections.

## 1. Introduction

Nosocomial infections are one of the main problems of modern healthcare. They are present in almost all hospitals, cause significant harm to the health of patients and bring about large economic losses. According to the World Health Organization, around ten million hospitalized patients worldwide die or become disabled every year due to poor medical practices in handling nosocomial infections. According to rough estimates, in developed countries, from 5 to 10% of patients in medical institutions suffer from nosocomial infections, in developing countries their number can reach 25% [1,2,3]. Nosocomial infections increase the duration of the inpatients’ hospital stay by two to three times, the cost of treatment by three to four times and the risk of death by five to seven times [4]. Direct financial losses from nosocomial infections, according to the WHO, annually amount to approximately 7 billion euros in Europe and 6.5 billion dollars in the United States [5].

The observed escalation of the problem of nosocomial infections is connected to the global spread of antibiotic resistance among microorganisms which results from uncontrolled and injudicious use of antibacterial drugs both in medicine and in various sectors of the food industry and agriculture [6,7]. According to the WHO, the growing bacterial resistance to antibiotics may devalue many of the achievements in medicine and epidemiology made to fight infectious diseases in the 20th century.

One of the main sources of nosocomial infections are microorganisms of the Enterobacteriaceae family, among which pathogenic strains of *E. coli* are of particular importance.

*E. coli* is a widespread commensal Gram-negative bacterium found in the gastrointestinal tract of humans and many warm-blooded animals. However, in the total mass of safe *E. coli* strains, there are pathogenic serotypes of *E. coli* that can cause infections of the intestinal tract and extraintestinal pathogenic E. coli (ExPEC). ExPEC can cause both community-acquired and hospital-acquired infections, such as urinary tract, soft tissue and central nervous system infections, and can lead to peritonitis and pneumonia [8,9,10,11,12]. Every year, hundreds of millions of people around the world suffer from diseases induced by pathogenic strains of *E. coli* [13,14]. Such strains are of particular danger to hospitalized patients with weakened immune systems. Nosocomial infections caused by *E. coli* can lead to the development of acute bacteremia and sepsis, cause organ failure and even death [15,16].

*E. coli* can be divided into eight main phylogenetic groups: A, B1, B2, C, D, E, F, I. Non-pathogenic, commensal E. coli strains that are the main representatives of the mammalian gastrointestinal tract belong to groups A and B1. Pathogenic *E. coli* strains, causative agents of intestinal infections, belong to groups A, B1, D. ExPEC mainly belong to groups B2 and D. *E. coli* strains of group E are closely related to group D, and those of group F to group B2. *E. coli* strains that are phenotypically the same but have diverse genomes are assigned to a separate branch I [17,18].

Uropathogenic strains of *E. coli* (UPEC), which belong to ExPEC, are responsible for up to 50% of cases of nosocomial urinary tract infections [19]. This is due to the systematic use of catheters on which bacteria reproduce and form UPEC biofilms leading to infection of patients [20]. UPEC infections are responsible for approximately 40,000 deaths and at least $2.6 billion in healthcare costs in the United States alone [21]. The isolated UPECs have diverse phenotypes and genotypes with different virulence factors: adhesins, toxins, iron acquisition systems, and defense against host immunity. In addition, nosocomial strains of UPEC are multidrug resistant, insensitive to first, second, and third generation antibiotics, which greatly complicates the fight against these pathogens using traditional methods [22].

The most common causative agents of Gram-negative bacterial neonatal meningitis are ExPEC strains of the B2 phylogroup (*E.coli* K1) [23]. Mortality from this infection is 10–15%, and neurological complications develop in 30–50% of cases [24]. Infection of newborns with such strains of *E. coli* mainly occurs in hospitals through the use of nasogastric tubes for feeding. Bacterial biofilms with a high degree of resistance to antibacterial agents are formed on these feeder probes [25,26]. To cross the blood–brain barrier, *E. coli* strains must possess virulence factors enabling them to bind to, enter, and survive in meningeal cells. Presumably the genetic determinants of such virulence factors are ibeA, sfaS, cnf1, gimA and ompA [27].

*E. coli* strains that grow on the tubes for artificial lung ventilation (ALV) systems and form biofilms there, are one of the main causative agents of nosocomial pneumonia [28]. This disease affects between 7% and 40% of critically ill patients [29]. Mortality among such patients increases by an average of 6%, and the duration of their stay in intensive care units by 10–12 days [30]. Most ExPEC strains isolated from ventilators belong to the B2 phylogroup and possess the sfa/foc, papGIII, hlyC, cnf1, and iroN virulence genes and resistance to amoxicillin, amoxicillin/clavulanic acid, aztreonam, ciprofloxacin chloramphenicol, and colistin [31].

In addition, constant contact with humans has led a large number of *E. coli* strains to develop resistance to almost all known classes of antibiotics. *E. coli* serotypes such as O157, O26, O45, O103, O111, O121 and O145 are known to be multidrug resistant and are one of the main causes of foodborne infections [32,33].

The widespread treatment of intestinal infections with first-line antibiotics, such as co-trimoxazole, amoxicillin, and amoxicillin in combination with clavulanic acid, has brought about the development of resistance to these drugs in *E. coli* [34]. The use of relatively new classes of broad-spectrum antibiotics, fluoroquinolones and cephalosporins, has led to the development of β-lactamase activity in *E. coli*, which also enables it to successfully resist these classes of antibiotics [35,36,37].

The situation is further complicated by the fact that at present, there is a reduction in funding for the development of new antibiotics and a subsequent decrease in the research activity. If this trend persists, it will eventually lead to the exhaustion of available options for combating resistant bacterial infections [38].

In such conditions, the danger of resistant nosocomial infections is becoming rampant, and research to find alternative ways to combat them is becoming increasingly relevant. Bacteriophages are considered the most promising alternative to antibiotics.

Bacteriophages are viruses that infect bacteria. Given the nature of bacteriophages as natural enemies of bacteria, their use as therapeutic agents to fight bacterial infections began shortly after their discovery by Felix D’Herelle [39]. However, after antibiotics were discovered and introduced into clinical practices, the interest in bacteriophages as a means of combating pathogenic bacteria declined sharply. Until the 1990s, the clinical use of bacteriophages was practiced mainly in Russia, Georgia and Poland [40,41,42].

But due to the global spread of multidrug resistance, the interest in bacteriophages as antibacterial agents and the related research activities have increased significantly over the past twenty years [43,44]. The advantage of bacteriophages is that they are very widespread and are present in all known ecosystems; according to rough estimates, their numbers on Earth reach 10^30^ viral particles, which makes them a virtually unlimited source for the search and isolation of new strains [40,45]. In addition, bacteriophages have a high specificity, which allows preservation of beneficial microflora when they are used to combat pathogens. Additionally, they are non-toxic and harmless for patients, possess the ability to self-replicate in host bacteria, and are able to penetrate into biofilms and lyse them [46,47,48].

Bacteriophages are used in agriculture to combat bacterial infections of cultivated plants and animals, and in medicine for disinfection of diagnostic equipment when chemical and thermal sterilization methods cannot be applied [41,49,50,51,52,53,54]. There are many examples of the successful use of bacteriophages as antibacterial agents [44,55].

Manohar P. et al. isolated and described the myPSH1131 bacteriophage that lysed 31 strains of pathogenic *E. coli*. In vivo experiments on the model of *G. mellonella* larvae showed that a single introduction of myPSH1131 phage to *E. coli* infected larvae increased their lifespan from 24 to 36 h. When the phage was administered three or four times, the mortality reduced to zero, confirming the complete eradication of the *E. coli* [50].

Montso P.K. et al. isolated eight bacteriophages from cattle feces that were able to infect a wide range of *E. coli* strains isolated from the environment. The isolated phages possessed high lytic activity and stability which made them potential agents for phage therapy [14].

Korf I. et al. showed the ability of a phage cocktail consisting of six previously isolated bacteriophages to lyse *E. coli* biofilms. Their research showed that unlike the use of single bacteriophages, the use of a phage cocktail prevented the development of phage resistance in *E. coli* strains [46].

But the use of bacteriophages in human therapy requires a cautious approach. Despite certain advantages of phage therapy, there are a number of negative factors that must be taken into account [56]. First, due to the high specificity of phages to their hosts, mass treatment of just one type of pathogenic bacteria requires an extensive collection of various bacteriophages to ensure the availability of the most effective lytic strain in each specific case. Secondly, not all the phages can be used for therapeutic purposes: it is not advisable to use bacteriophage strains with lysogeny or strains containing genes encoding bacterial toxins and genes conferring antibiotic resistance to bacteria. Thirdly, bacteria can develop resistance to bacteriophages, which must be taken into consideration during long-term phage therapy.

Therefore, successful introduction and application of phage therapy in mass medicine, requires constant replenishment of the collections with safe and effective strains of lytic bacteriophages. It is also necessary to study and develop different methods of using bacteriophages in the treatment of bacterial infections. One of the widely used methods in phage therapy is the use of mixtures consisting of different strains of bacteriophages—phage cocktails. This method can significantly reduce the development of bacterial resistance to phages, as well as increase the applicability of phage preparations against various bacterial infections [57,58].

The purpose of this study was to isolate bacteriophages capable of lysing clinical strains of *E. coli* from the environment, study their biological properties and investigate the properties of a cocktail consisting of the isolated phages.

## 2. Materials and Methods

### 2.1. Bacterial Strains

Nine different strains of *E. coli* (Table 1) were used in the study. A total of eight of them were clinical strains provided by the bacterial laboratory of the Central Clinical Hospital JSC in Almaty. Information on antibiotic resistance of these eight strains obtained by using the microbiological analyzer VITEK 2 (bioMérieux) was also provided by the JSC. One strain of *E. coli* 15,597 was purchased from ATCC.

### 2.2. Isolation of Bacteriophages

Bacteriophages were isolated from wastewater samples obtained from a sewage collector of Shymkent, Kazakhstan and from sewage disposal fields of Konaev, Kazakhstan.

The wastewater samples were obtained from the depth of 15–20 cm using sterile 500 mL containers. The aqueous samples were transported and stored at a temperature of 4–8 °C in opaque packaging and were not exposed to direct sunlight. To inactivate pathogenic microorganisms, chloroform was added to the aqueous samples at the concentration of 0.1% and allowed to sit for 24 h at a temperature of 4–8 °C. The samples were then filtered through a paper filter and through a 0.45 μm bacterial filter (Sartolab^®^ RF 500, Sartorius, Goettingen, Germany). Then the samples were stored in sterile conditions in a dark place at a temperature of 4–8 °C.

The aqueous samples were enriched with bacteriophages using the methods described by Merabishvili M. et al. [59]: 1 mL of 10× nutrient broth (Nutrient Broth, Himedia, Thane, India), 1 mL of indicator bacteria suspension with a turbidity of 1 McFarland (10^8^ CFU/mL) and 100 µL of 1% MgSO_4_ were added to 10 mL of the prepared filtrate. The mixture was cultivated for 18–24 h at 37 °C with aeration. The resulting phage lysate was centrifuged, the obtained supernatant was filtered through 0.45 µm bacterial filters (Nalgene Syringe Filter, Thermo Scientific, MA, USA).

### 2.3. Determination of Bacteriophage Titer

Bacteriophages were titrated using the double-layer agar technique (DLA or Gratia method) [60]. 1 mL of the sample was mixed with 2.5 mL of 0.7% nutrient agar (Nutrient Agar “Himedia”, India) and 1 mL of indicator bacteria suspension with a turbidity of 1 McFarland containing 10^8^ CFU/mL. The prepared mixture was poured into Petri dishes on the surface of a previously prepared 2% nutrient agar and incubated for 18–24 h at 37 °C. The same mixture was used as a control but with 1 mL of sterile phosphate buffer instead of the aqueous sample. At the end of the incubation period, single PFUs were observed.

### 2.4. Pure Bacteriophages Isolation

Pure lines of bacteriophages were isolated using the methods described by Montso P.K., et al. [14]. After PFUs were formed on the double layer of agar, a clear lysis zone of a certain morphotype was selected at the maximum dilution of the phage lysate, picked up from the agar and placed in 5 mL of sterile nutrient broth, incubated for 2 h at 37 °C, after which the procedure of enrichment with bacteriophages was carried out, and again titrated on double-layer agar. Subsequently, newly formed PFUs were selected at the maximum dilution and a clear lysis zone of the same morphotype was picked up from the agar to be seeded again following the above method. This complete cycle was repeated at least 3 times until PFUs of only one morphotype were obtained.

### 2.5. Bacteriophage Propagation

40 mL of sterile nutrient broth was poured into sterile 50 mL test tubes, then 500 µL of propagated bacteriophage and 5 mL of a 24 h culture of host bacteria were added and cultivated for 24 h at 37 °C. After the cultivation, the phage lysate was purified by centrifugation, resulting supernatant was filtered through bacterial filters, and the precipitate was discarded.

### 2.6. Concentration of Bacteriophages by Ultracentrifugation

The bacteriophages were sedimented from the phage lysate by centrifugation at 100,000× *g* for 90 min at 4 °C in an Avanti J-30i centrifuge (Beckman Coulter, CA, USA). The resulting bacteriophage sediment was dissolved in a minimum volume of PBS (1 mL) [61].

### 2.7. Transmission Electron Microscopy Analysis

The structure of the isolated bacteriophages was studied by transmission electron microscopy (TEM) at an instrumental magnification of 60–120 thousand times and a voltage of 100 kV using a JEM-2100 microscope (JEOL, Tokyo, Japan). 10 µL of each purified phage lysate was spotted on formvar coated copper grid (Polysciences, Inc., Hirschberg an der Bergstrasse, Germany) for 5 min. Then, 3% phosphotungstic acid (pH 6.8) was used to stain the samples, and excess solution was removed.

### 2.8. Phage DNA Isolation and Genome Sequencing

Genomic DNA was isolated from the phage lysate according to the PureLink viral DNA/RNA minikit manual (Thermo Fisher Scientific, MA, USA). The DNA library was prepared using the Nextera XT DNA Sample Preparation Kit (Illumina, CA, USA). Whole genome sequencing was performed using the Illumina MiSeq sequencing platform using kit v3.

### 2.9. Bioinformatics Analysis

Low quality reads were filtered out and adapters trimmed using Trimmomatic from the Genome Detective tool [62,63]. Genomes were assembled from paired reads using SPAdes 3.12.0 [64]. The average read length after trimming was 286 bp. The genomes were assembled to an average depth of more than 1000 [65]. The physical ends of the viral genomes were identified by comparing the total length coverage of the genomes of the studied viruses and closely related viruses in the NCBI database using nBLAST [66], namely Escherichia phage APCEc01 (NC_029091) with 100% query coverage and 97.97% identity for the vB_EcoM_SCS57 phage, Escherichia phage VB_EcoS-Golestan (NC_042084) with 100% query coverage and 99.51% identity for vB_EcoS_SCS44 phage, Escherichia phage vB_EcoS_ACG-M12 (JN986845.1) with 100% query coverage and 93.36% identity for vB_EcoS_SCS31 phage, Escherichia phage ECML-134 (JX128259.1) with 100% query coverage and 98.17% identity for vB_EcoM_SCS4 phage, Escherichia phage NTEC3 (OK539620.1) with 100% query coverage and 96.7% identity for vB_EcoS_SCS92 phage, Escherichia phage vB_EcoP_F (NC_047808.1) with 100% query coverage and 98.76% identity for the vB_EcoP_FFK3 phage. Then, closely related sequences were imported into Geneious Prime to generate a phylogenetic tree using the following default parameters: CLUSTAL-W aligner and the Geneious Tree Builder. Comparisons of multiple phage genome sequences were visualized using the BLAST Ring Image Generator (BRIG) with default settings [67]. Open-reading frames were predicted using the ORF finder tool (https://www.ncbi.nlm.nih.gov/orffinder/; accessed on 1 September 2022) using methionine and alternative initiation codons as start codons. The putative coding sequences (CDSs) were predicted using BLASTp of the predicted ORFs against the NCBI non-redundant protein sequences (nr) database. To increase the confidence in the predicted ORFs and CDSs, they were compared to those predicted through PHASTER [68]. The genome maps of the investigated bacteriophages were generated using Geneious Prime software.

### 2.10. Assessment of the Lytic Activity Level of Bacteriophages

The level of lytic activity of isolated bacteriophages was determined by their co-cultivation with indicator bacteria in microplates. A series of sequential 10-fold dilutions of the initial phage lysate was prepared to obtain the values of multiple infection (multiplicity of infection (MOI)) in the range from 10^−7^ to 1. 150 μL of LB, 50 μL of a phage-containing sample and 50 μL of a bacterial suspension containing 10^8^ CFU/mL (turbidity of 1 McFarland) were put into the wells of the microplate. Samples containing 50 µL of sterile LB instead of bacteriophages were used as a control for the growth of the bacterial test culture. As a negative control, a sample containing only 200 µL of sterile LB and 50 µL of the original phage lysate was used. Cultivation was carried out at 37 °C in an Infinite^®^ 200 PRO plate reader (Tecan, Männedorf, Switzerland). The dynamics of bacterial growth was assessed by the change in the optical density of the suspension at 600 nm. Microplates were scanned every 30 min during the entire cultivation period [69]. Each experiment was repeated 3 times.

### 2.11. Host Range Determination

Host ranges were determined by co-cultivation of isolated bacteriophages with various *E. coli* strains in microplates. The titer of the bacteriophages was 10^8^ PFU/mL, and the titer of the bacteria was 10^8^ CFU/mL. Cultivation was carried out in an Infinite^®^ 200 PRO plate reader (Tecan, Mennedorf, Switzerland) at 37 °C as described above. Each experiment was repeated 3 times.

### 2.12. Development of a Bacteriophage Cocktail

To form the phage cocktail, equal volumes of the phage lysates of the isolated strains were mixed, and the resulting cocktail was passed through a 0.45 μm bacterial filter. The concentration of each bacteriophage strain was 10^8^ viral particles per mL.

### 2.13. Investigation of the Bacteriophage Cocktail Stability after Storage

The bacteriophage cocktail was kept for 8 months using three different methods of storage: (1) in sterile conditions at room temperature, (2) in sterile conditions at 4–8 °C, (3) in a freeze-dried state with the addition of a stabilization medium (0.8 M sucrose solution). The stability of the biopreparations was determined based on their lytic activity. Lytic activity was assessed every month for each storage method by co-cultivating the phage cocktail with a bacterial test culture in a microplate.

### 2.14. Statistics and Graphic Display of Results

The statistical analysis of the results was performed using Statistica 10.0 and Microsoft Excel. Microsoft Office was then used to create tabular and graphic representation of the findings. Student’s unpaired *t*-test was used to evaluate the difference between the test sample and the untreated control. Values for all of the parameters were expressed as the mean ± the standard deviation (SD). *p*-values less than 0.05 were considered statistically significant.

## 3. Results

### 3.1. Isolation and Characterization of E. coli Bacteriophages

**Figure 1:**
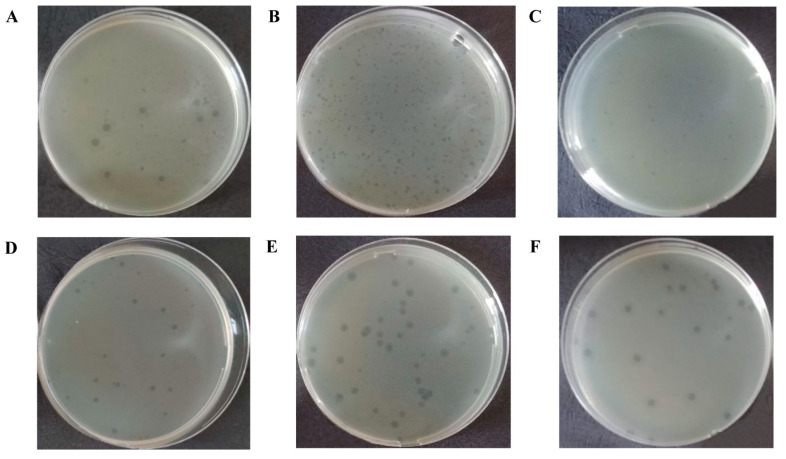
The plaques of isolated bacteriophages: vB_EcoM_SCS4 (**A**), vB_EcoS_SCS44 (**B**), vB_EcoM_SCS57 (**C**), vB_EcoS_SCS92 (**D**), vB_EcoS_SCS31 (**E**), vB_EcoP_FFK3 (**F**).

Six strains of bacteriophages were isolated from the wastewater samples by successive selective passages on double agar. *E. coli* 444, *E. coli* 3957, *E. coli* 4231, *E. coli* 3992 strains were used to isolate one bacteriophage each, and E. coli 753 was used to isolate two bacteriophages. Five strains of bacteriophages were isolated from the samples obtained from the sewage collector of Shymkent, Kazakhstan. One strain of bacteriophage came from the samples obtained from the sewage disposal fields of Konaev, Kazakhstan. The isolated bacteriophages were designated as: vB_EcoM_SCS4, vB_EcoS_SCS44, vB_EcoM_SCS57, vB_EcoS_SCS92, vB_EcoS_SCS31, vB_EcoP_FFK3. When infected with the isolated bacteriophages, the initial host strains produced plaques of 2–4 mm in diameter with a halo of up to 8 mm (Figure 1, Table 2).

**Figure 2:**
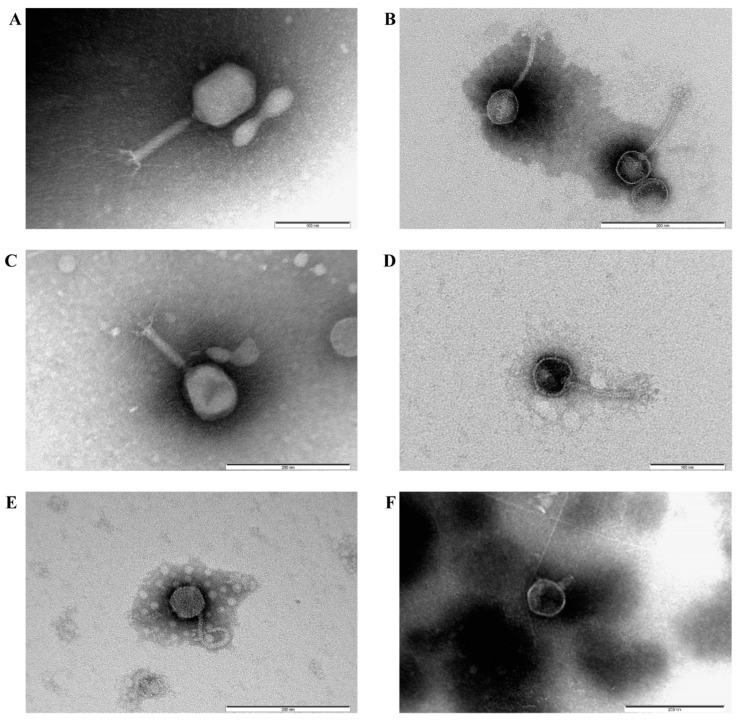
Electron microscopy of bacteriophages *E. coli*: vB_EcoM_SCS4, ×120,000 (**A**), vB_EcoS_SCS44, ×100,000 (**B**), vB_EcoM_SCS57, ×100,000 (**C**), vB_EcoS_SCS92, ×120,000 (**D**), vB_EcoS_SCS31, ×100,000 (**E**), vB_EcoP_FFK3, ×100,000 (**F**).

Electron microscopy showed (Figure 2) that all the isolated bacteriophages were 150–200 nm long, had heads and tails of different lengths, and hence belonged to the *Caudoviricetes* class of tailed bacteriophages. vB_EcoM_SCS4, vB_EcoM_SCS57 had elongated hexagonal heads and tails surrounded by a contractile sheath, which morphologically characterized them as representatives of the myoviruses group (Figure 2A,C). Phages vB_EcoS_SCS44, vB_EcoS_SCS92, vB_EcoS_SCS31 had hexagonal heads and elongated flexible non-contractile tails and belonged to the siphoviruses group (Figure 2B–E). The vB_EcoP_FFK3 phage had a hexagonal head and a short tail characteristic of the podoviruses group (Figure 2F).

**Figure 3:**
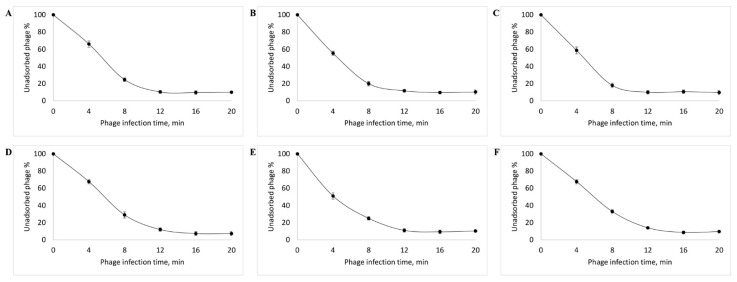
Adsorption rate of the vB_EcoM_SCS4 (**A**), vB_EcoS_SCS44 (**B**), vB_EcoM_SCS57 (**C**), vB_EcoS_SCS92, (**D**), vB_EcoS_SCS31 (**E**), vB_EcoP_FFK3 (**F**) bacteriophages.

The study of virus adsorption revealed that, in case of all the isolated bacteriophages, about 90% of the virus particles were adsorbed into the host cell within 10 min (Figure 3).

**Figure 4:**
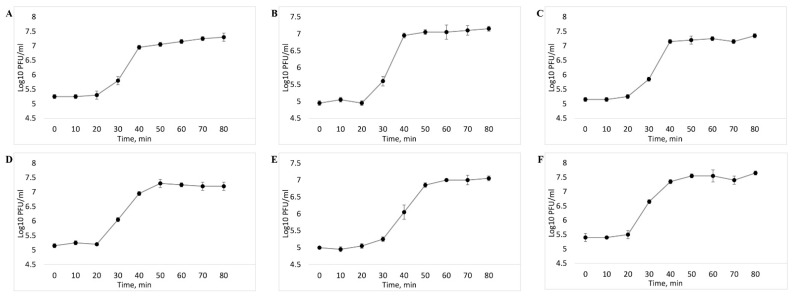
One-step growth curve of the vB_EcoM_SCS4 (**A**), vB_EcoS_SCS44 (**B**), vB_EcoM_SCS57 (**C**), vB_EcoS_SCS92, (**D**), vB_EcoS_SCS31 (**E**), vB_EcoP_FFK3 (**F**) bacteriophages.

One-step growth curve made it possible to establish that the latent period of five out of the six investigated bacteriophages averaged 20 min. It was followed by a rise lasting from minute 20 to minute 40, after which it reached a plateau (Figure 4A–D,F). vB_EcoS_SCS31 phage had the latent phase of 30 min, followed by a burst size period between minute 30 and minute 50 (Figure 4E).

### 3.2. Bioinformatics Analysis and Characterization of the Bacteriophages’ Genome

**Figure 5:**
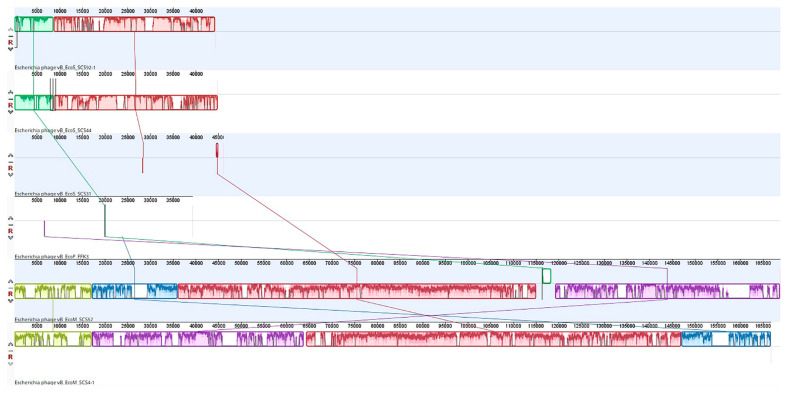
Alignment of the complete genomes of the isolated phages.

After genome sequencing of six isolated bacteriophages, multiple alignment was carried out using the Mauve program (Figure 5), which clearly showed that the studied viruses belonged to four groups.

**Figure 6:**
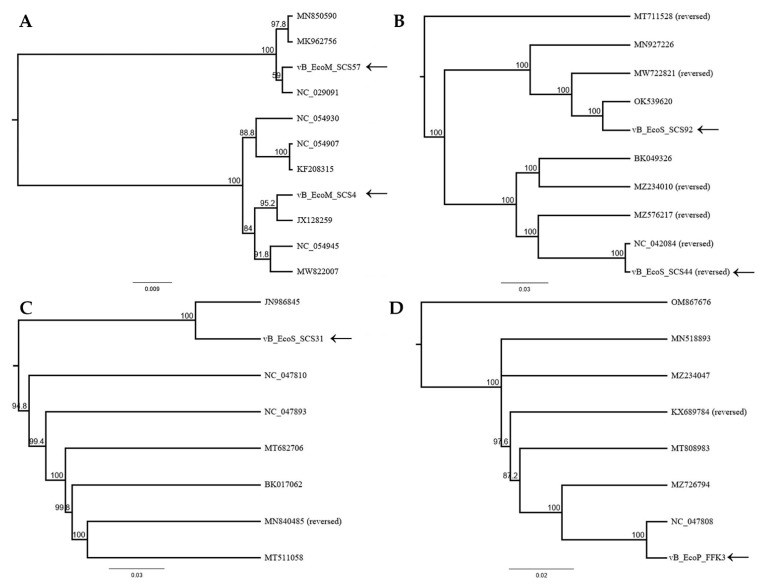
Phylogenetic trees of the studied phages: *Straboviridae* family (**A**), *Guernseyvirinae* subfamily (**B**), *Drexlerviridae* family (**C**), *Autographiviridae* family (**D**).

The studied bacteriophages possessed a dsDNA genome and belonged to four families of the *Caudoviricetes* class: two of them belonged to the *Straboviridae* family (vB_EcoM_SCS4, genome length of 166,783 bp and vB_EcoM_SCS57, genome length of 168,771 bp), two to the *Guernseyvirinae* subfamily(vB_EcoS_SCS44, genome length of 44,829 bp and vB_EcoS_SCS92 genome length of 44,240 bp), one to the *Drexlerviridae* family (vB_EcoS_SCS31 genome length of 46,054 bp), and one to *Autographiviridae* family (vB_EcoP_FFK3, genome length of 39,300 bp). The complete genomes of the vB_EcoM_SCS4, vB_EcoM_SCS57, vB_EcoS_SCS44, vB_EcoS_SCS92, vB_EcoS_SCS31, vB_EcoP_FFK3 phages were sequenced and deposited into the NCBI GenBank database (GenBank Acc. No. ON506924, OM960734, ON001686, ON548432, ON081052, ON548433, respectively). BLASTn analysis and construction of phylogenetic trees using major capsid protein sequences of the investigated phages and closely related phages from the NCBI database confirmed that the genomes of the isolated bacteriophages were different from one another, and belong to the corresponding families of the *Caudoviricetes* class (Figure 6).

In addition, we carried out a comparative analysis of the whole-genome sequences of the investigated phages with the same phages used in the phylogenetic analyses. Similar to the results of the major capsid protein analysis, phages vB_EcoM_SCS57, vB_EcoM_SCS4, vB_EcoS_SCS92, vB_EcoS_SCS44, vB_EcoS_SCS31 and vB_EcoP_FFK3 grouped with the phages Escherichia phage APCEc01 (NC_029091.1), Escherichia phage ECML-134 (JX128259), Escherichia phage NTEC3 (OK539620.1), Escherichia phage VB_EcoS-Golestan (NC_042084), Escherichia phage vB_EcoS_ACG-M12 (JN986845.1), Escherichia phage vB_EcoP_F (NC_047808.1), respectively, and shared lower similarity with other phages (Figures S1–S6).

Open reading frames (ORF) prediction using the standard genetic code identified 272 putative protein-coding genes (Table 3). The 123 predicted proteins had assigned functions in the vB_EcoM_SCS57 phage genome. This phage had 231 open reading frames on the leading strand and 41 ORFs on the complementary strand. In addition, 149 genes encoding hypothetical proteins and 123 functional genes were identified in vB_EcoM_SCS57, of which 84 ORFs were located on the leading strand and 39 ORFs on the complementary strand. vB_EcoM_SCS4 had 269 genes encoding 47 functional proteins, with 28 ORFs on the leading strand and 19 ORFs on the complementary strand.

In the genomes of vB_EcoS_SCS44 and vB_EcoS_SCS92 phages, 78 and 72 putative protein-coding genes, respectively, were identified. In the vB_EcoS_SCS44 phage sequence, 34 of the predicted proteins had assigned functions with 22 located on the leading strand and 12 on the complementary strand, while in the bacteriophage vB_EcoS_SCS92 genome, out of 72 predicted proteins, 28 had assigned functions (20 on the complementary strand, and 8 on the leading strand).

The vB_EcoS_SCS31 phage genome consisted of 78 putative genes, 54 of which encoded hypothetical proteins, and 24 proteins with assigned functions, 20 of which were located on the leading strand and 4 on the complementary one.

Open reading frame prediction in the whole-genome sequence of the vB_EcoP_FFK3 phage revealed 48 genes, 29 of which encoded proteins with assigned functions, all located on the leading strand.

The functional genes of all six phages are presented on genetic maps (Supplementary Figures S7–S12). Functional proteins in the studied phages were assigned functions such as cell lysis proteins, DNA replication/transcription/repair proteins, structural proteins, and DNA packaging proteins.

### 3.3. Determination of the Level of Lytic Activity

The level of lytic activity of isolated bacteriophages was assessed by the ability of different doses of viruses to suppress the growth of the same number of bacteria (MOI 10^−7–1^). The titer of the initial samples of bacteriophages was 10^8^ viral particles per 1 mL. For each studied bacteriophage, the original bacterial host strain was used as a test culture. The dynamics of bacterial growth during co-cultivation with the bacteriophage was determined in microplates using a multichannel spectrophotometer for 6 h.

**Figure 7:**
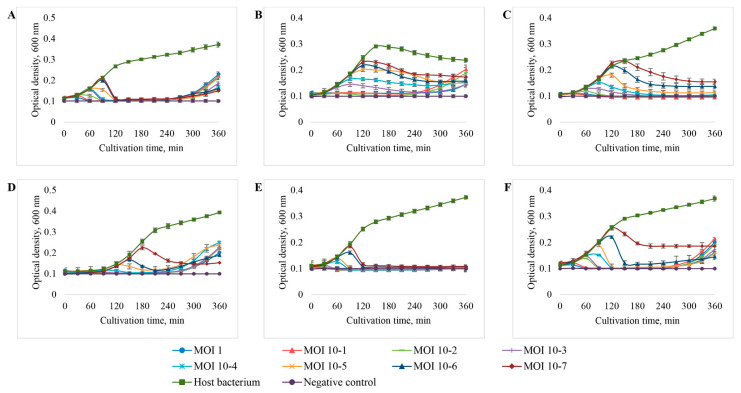
The effect of *E. coli* bacteriophages at MOI from 10^−7^ to 1 on the growth of the host bacterium: (**A**) vB_EcoM_SCS4, (**B**) vB_EcoS_SCS44, (**C**) vB_EcoM_SCS57, (**D**) vB_EcoS_SCS92, (**E**) vB_EcoS_SCS31, (**F**) v_BEcoP_FFK3.

As a result of the study, it was found (Figure 7, Table 4) that the vB_EcoM_SCS4 and vB_EcoS_SCS31 bacteriophages possessed the highest lytic activity and were able to completely suppress the growth of bacterial cultures at all the MOIs used (Figure 7A,E). Bacteriophages vB_EcoS_SCS92 and vB_EcoP_FFK3 completely suppressed the growth of bacterial cultures at MOI 10^−6^ to 1, while the inhibition of bacterial growth at MOI 10^−7^ was approximately at the level of 70 and 60%, respectively (Figure 7D,F). Bacteriophage vB_EcoM_SCS57 completely suppressed the growth of the bacterial test culture at MOI 10^−5^ to 1, while the growth of the bacterial culture at MOI 10^−6^ and 10^−7^ was inhibited by 85 and 80%, respectively (Figure 7C). Bacteriophage vB_EcoS_SCS44 had the lowest lytic activity which completely suppressed the growth of the bacterial test culture at MOI 10^−3^ to 1, while the inhibition of bacterial growth at MOI 10^−7^ to 10^−4^ made 50–70% (Figure 7B).

The most rapid inhibition of the growth of bacterial cultures was recorded at MOI values of 1. At MOI values of 1, all the isolated bacteriophages showed complete inhibition of host bacteria growth even after the first 30 min of cultivation, and after an hour of cultivation, the optical density of experimental samples decreased to the level of the negative control.

After 4.5 h of co-cultivation of host bacteria with bacteriophages vB_EcoM_SCS4, vB_EcoS_SCS44, vB_EcoS_SCS92, v_BEcoP_FFK3, re-growth of bacterial cultures was recorded. In the samples with vB_EcoM_SCS57 and vB_EcoS_SCS31 bacteriophages, no re-growth of bacterial cultures was observed during the entire cultivation period.

Given their maximum efficiency, MOI values of 1 were used for further studies of the isolated bacteriophages.

### 3.4. Host Range Analysis

The range of hosts of isolated bacteriophages was determined on eight clinical *E. coli* strains—444, 753, 3957, 3992, 4231, 4238, 4320, 4325—and one *E. coli* strain 15,597 purchased from ATCC. The activity of phages in relation to the *E. coli* used was assessed by the changes in the growth dynamics of the bacterial cultures. Cultivation of bacteria with the addition of the studied phages was carried out for 6 h. The MOI value was 1 in all cases. The dynamics of bacterial growth was assessed by the change in the optical density of the culture medium at 600 nm.

**Figure 8:**
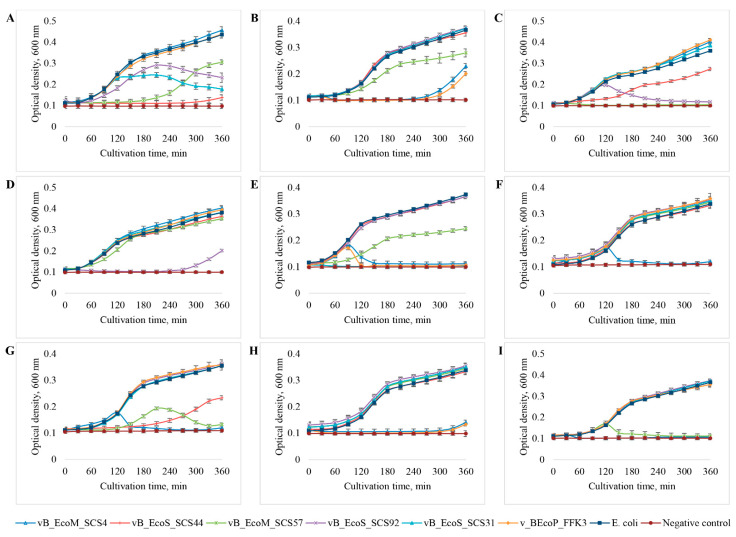
Growth dynamics of various strains of *E. coli*—444 (**A**), 753 (**B**), 3957 (**C**), 3992 (**D**), 4231 (**E**), 4238 (**F**), 4320 (**G**), 4325 (**H**), 15,597 (**I**) in the presence of the studied bacteriophages.

As a result of the study, it was found (Figure 8, Table 5) that the vB_EcoM_SCS4 bacteriophage had the widest range of hosts: it completely suppressed the growth of six out of nine *E. coli* strains used. The vB_EcoM_SCS57 bacteriophage also suppressed the growth of six *E. coli* strains, but the growth of *E. coli* 753 and 4231 was not completely suppressed by this bacteriophage—only by 30% and 50%, respectively. Bacteriophages vB_EcoP_FFK3, vB_EcoS_SCS92 and vB_EcoS_SCS44 were able to lyse three out of nine studied *E. coli* strains: 753, 4231 and 4325, while the vB_EcoP_FFK3 phage completely suppressed the growth of these three bacterial strains. The vB_EcoS_SCS92 phage completely inhibited the growth of *E. coli* 3957, 3992 and suppressed the growth of *E. coli* 444 by 40–50%. The vB_EcoS_SCS44 phage completely inhibited the growth of only the host bacterium (*E. coli* 444) and also partially suppressed the growth of *E. coli* 3957 and 4320 strains—by about 50%. Bacteriophage vB_EcoS_SCS31 had the minimum range of hosts: in addition to the host bacterium, it partially lysed *E. coli* 444.

### 3.5. Development of the Bacteriophage Cocktail and Determination of Its Efficiency and Spectrum of Lytic Activity against E. coli

The bacteriophage cocktail included all six isolated bacteriophages: vB_EcoM_SCS4, vB_EcoS_SCS44, vB_EcoM_SCS57, vB_EcoS_SCS92, vB_EcoS_SCS31 and v_BEcoP_FFK3.

To form the cocktail, each of the bacteriophage strains were added at the concentration of 10^8^ viral particles per ml. The total concentration of phages in the cocktail was 6 × 10^8^. The efficiency and activity spectrum of the phage cocktail were determined for nine previously used *E. coli* strains (444, 753, 3957, 3992, 4231, 4238, 4320, 4325, 15597). To achieve MOI 1, the concentration of bacterial cultures was 10^8^ cells per 1 mL, which corresponds to a turbidity of 1 McFarland. The activity of the phage cocktail was also assessed by the change in the optical density of the culture medium during 6 h of co-incubation with bacterial cultures.

**Figure 9:**
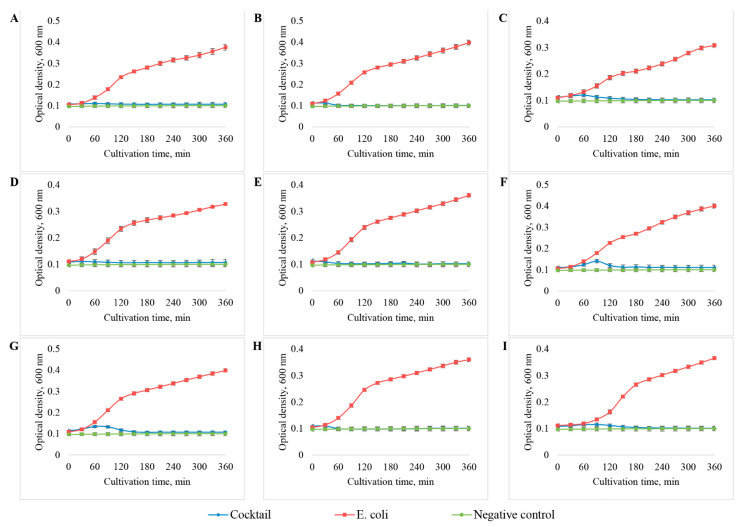
Growth dynamics of various *E. coli* strains: 444 (**A**), 753 (**B**), 3957 (**C**), 3992 (**D**), 4231 (**E**), 4238 (**F**), 4320 (**G**), 4325 (**H**), 15,597 (**I**) in the presence of the bacteriophage cocktail.

It was shown (Figure 9) that a cocktail formed from the isolated *E. coli* bacteriophages completely suppressed the growth of all the used *E. coli* cultures throughout the entire cultivation period.

### 3.6. Comparative Study of the Efficiency of E. coli Bacteriophage Cocktail Storage Methods

The *E. coli* bacteriophage cocktail was stored using three methods: (1) storage in sterile conditions at room temperature, (2) storage in sterile conditions at 4–8 °C, (3) storage in a freeze-dried state with the addition of a stabilization medium (0.8 M sucrose solution).

The storage period was 8 months. Storage efficiency was determined by the change in the level of lytic activity of the cocktail over the course of the storage time. The lytic activity of the cocktail was evaluated by the ability to 100% inhibit the growth of the bacterial test culture. The *E. coli* 15,597 strain obtained from ATCC was used as test culture. The cocktail was tested at eight different concentrations, from the initial to seven consecutive 10-fold dilutions. The turbidity of the bacterial suspension was adjusted to 1 McFarland standard before use, therefore, MOI values from 10^−7^ to 1 were obtained. The lytic activity of the cocktail was tested once a month throughout the entire storage period. Quantitative determination of bacterial growth during co-cultivation with the phage cocktail was made in microplates using a multichannel spectrophotometer for 6 h.

**Figure 10:**
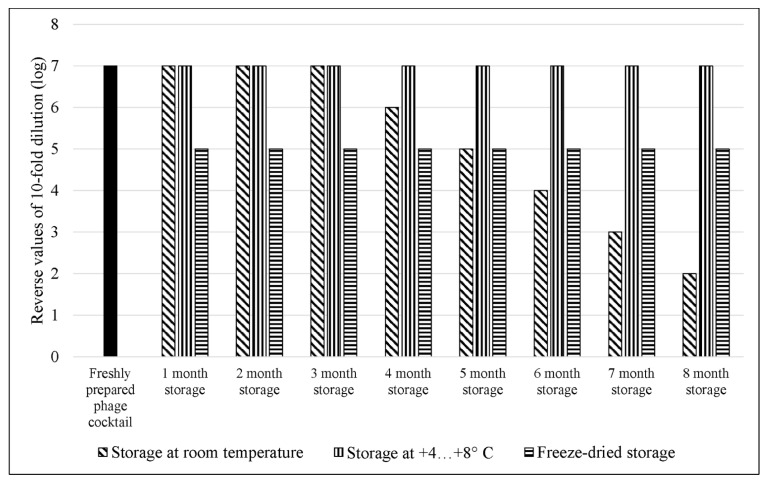
The concentration of viral particles in the *E. coli* bacteriophages cocktail required to completely inhibit the growth of the bacterial culture depending on the duration and method of storage. The *E. coli* 15,597 strain obtained from ATCC was used as the test culture. Each experiment was performed in triplicate.

The study showed (Figure 10) that a freshly prepared cocktail of anti-*E. coli* bacteriophages inhibited the growth of the bacterial test culture by 100% at a concentration of 10 viral particles per 1 mL (MOI of 10^−7^). The same lytic activity of the cocktail was observed after 8 months of storage in sterile conditions at 4–8 °C. The storage of the bacteriophage cocktail in sterile conditions at room temperature allowed it to maintain its maximum lytic activity during the first 3 months. After that, the activity decreased by 1 log with each month of storage, and after 8 months of storage at room temperature, 100% growth inhibition of the bacterial test culture was observed at MOI 10^−2^. After freeze-drying, the lytic activity of the cocktail reduced by 2 log, and complete lysis of the bacterial culture was recorded at MOI 10^−5^; the freeze-dried cocktail then maintained this level of lytic activity throughout the entire storage period.

## 4. Discussion

There are many examples of successful clinical use of phage therapy for combating bacterial infections, including their antibiotic-resistant forms [44,70]. But it is necessary to take into account the high specificity of bacteriophages to their hosts. On the one hand, it is an advantage since during phage therapy only pathogenic target bacteria are lysed and beneficial or neutral microflora do not suffer. But on the other hand, it necessitates the selection of the most effective bacteriophages or a phage cocktail for each specific case of bacterial infection. In this connection, it is necessary to constantly search for and isolate new lytic bacteriophages, replenish and to expand the existing collections.

The aim of this study was to isolate bacteriophages capable of lysing clinical strains of *E. coli*. Urban sewage wastewater is the richest source of human variants of *E. coli* and their bacteriophages, so it was decided to isolate the bacteriophages from such reservoirs, especially since this approach had repeatedly produced positive results in various studies [71,72,73,74]. As a result, six bacteriophages capable of lysing clinical strains of *E. coli* were isolated on five clinical strains of *E. coli*.

Transmission electron microscopy showed that all the isolated phages could be classified as members of the *Caudoviricetes* class, typical bacteriophages that infect *E. coli*. Their morphological features—hexagonal heads and tails of different lengths—were characteristic of viruses of this class [75]. Phages vB_EcoM_SCS4 and vB_EcoM_SCS57 had an elongated hexagonal head with a diameter of approximately 90–100 nm and a contractile tail of approximately 100–110 nm in length; in both cases, the basal plate with short tail filaments was clearly visible. The images of the vB_EcoM_SCS57 phage clearly show long tail filaments. Phages of such structure belong to the myoviruses group, the most typical and studied representatives of *E. coli* bacteriophages [42]. Bacteriophages vB_EcoS_SCS44, vB_EcoS_SCS92 and vB_EcoS_SCS31 had a 50–60 nm hexagonal head and a 100–150 nm long non-contractile tail. The vB_EcoS_SCS44 and vB_EcoS_SCS92 phages had well-defined side tail protein filaments in the images, and the vB_EcoS_SCS31 phage had a flexible tail. Such structural features correspond to the phages of the siphoviruses group, whose representatives are typical *E. coli* viruses and are often isolated on this type of bacteria [76,77]. Bacteriophage vB_EcoP_FFK3 had a hexagonal head of about 100 ± 0.5 nm and a short non-contractile tail of about 50 ± 0.5 nm in length—the structure typical for phages of the podoviruses group, which are also representatives of enterobacteria viruses and are isolated on *E. coli* cultures [78,79].

All the bacteriophages isolated in this study possessed a high adsorption rate (8–10 min) which ensured their rapid reproduction [80]. At a high adsorption rate, phages meet the host more often and attack it faster; this leads to rapid lysis and thereby increases the therapeutic effect of such bacteriophages [81].

One-step growth experiments showed that all the isolated phages have a short latent period (20–30 min) and an average burst size of more than 100 PFU per cell. The obtained data confirmed the high level of reproduction of the studied phages and, hence, their capability to quickly lyse host bacteria and massively spread among them [58]. Bacteriophages with a short latency period and a high burst size are considered highly lytic and are most preferred for uses in phage therapy.

The results of whole genome sequencing of isolated bacteriophages made it possible to clarify their taxonomic affiliation. It was shown that all isolated bacteriophages belonged to the *Caudoviricetes* class [75]. The data obtained confirm the results of TEM; the structure of the isolated phages fully corresponds to their taxonomic affiliation.

The prediction of open reading frames made it possible to establish that the genome of the isolated bacteriophages did not encode genes of lysogeny factors (genes of transposases, integrases, prophages, etc.) and antibiotic resistance [82].

In determining the level of lytic activity, it was found that the isolated bacteriophages were able to completely lyse their host bacteria at MOI 10^−7^–10^−3^ and inhibit the growth of bacteria by at least 50% at minimum concentrations. The ability of the phages to completely inhibit the growth of bacterial cells for at least 4 h at such low concentrations indicates their high antibacterial activity.

In addition, when performing these experiments, it was found that in the case of vB_EcoM_SCS4, vB_EcoS_SCS44, vB_EcoS_SCS92 and v_BEcoP_FFK3, bacterial culture re-growth was recorded after an average of 4.5 h of cultivation. This fact indicates that the lysis was not 100% and there remained some bacteria resistant to phage infection which gradually multiplied or new resistant strains had time to form, as described in the studies of Kim M et al. and Kocharunchitt C et al. [83,84]. Lower concentrations of phages either did not lead to re-growth of bacteria within 6 h of observation (vB_EcoS_SCS44, _BEcoP_FFK3), or bacterial growth was detected at a later stage (vB_EcoM_SCS4, vB_EcoS_SCS92). This can mean that the infection was weaker, and, as a consequence, resistant bacteria accumulated more slowly [85,86].

In the case of bacteriophages vB_EcoM_SCS57 and vB_EcoS_SCS31 bacteria re-growth was not recorded at any of the MOIs used. This can be explained either by insufficient incubation time to form resistance in bacteria and enable re-growth, or, in the case of vB_EcoS_SCS31 phage, by the actual 100% lysis of bacteria.

The high specificity of bacteriophages to their hosts requires an individual approach to each individual case and to each patient, which increases the time and cost of obtaining a stable positive effect in phage therapy of bacterial infections [87]. In this connection, it is necessary to determine the possible spectrum of hosts in therapeutic bacteriophages for their further prompt and effective use.

Studies performed to determine the host spectrum of isolated *E. coli* bacteriophages showed that none of the bacteriophage strains studied had the ability to lyse all the bacterial cultures used. Therefore, a phage cocktail was the most appropriate solution as it increased the spectrum of antibacterial activity.

vB_EcoM_SCS4 and vB_EcoM_SCS57 had the broadest host range among the studied bacteriophages. These phages are myoviruses: both membrane proteins of the porin family and lipopolysaccharide (LPS) nuclei can serve as secondary adsorption receptors for them, and this increases the number of bacteria they infect. In turn, only porin family proteins are adsorption receptors for siphoviruses, while podoviruses only bind to LPS nuclei [88]. Therefore, phages of these two groups potentially have a narrower spectrum of hosts.

With the mass spread of phage therapy, preparations with a broad host spectrum will be favored as this simplifies their application and reduces the production cost. The most proven method of increasing the spectrum of affected bacteria is developing cocktails consisting of bacteriophage strains of different families with different lytic activity [89,90].

In addition, the use of phage cocktails reduces or even precludes the probability of bacteria developing resistance to them during the period of therapy, which significantly increases its effectiveness [46,91,92].

A phage cocktail was formed from the isolated bacteriophages and its spectrum of activity on nine clinical strains of *E. coli* (444, 753, 3957, 3992, 4231, 4238, 4320, 4325, 15597) was determined. The results showed that the developed cocktail completely suppressed the growth of all the *E. coli* strains used during the entire period of cultivation. No secondary bacterial growth was detected, indicating the combined ability of the cocktail phages to overcome the resistance mechanisms of the lysed *E. coli* strains.

There are several hypotheses explaining such synergy of bacteriophages in the cocktail. Latka A. et al. in their studies suggest that co-infection with several phages involves the synthesis of various enzymes of polysaccharide depolymerization capable of damaging bacterial cell walls, facilitating phage access to the cytoplasmic membrane of the cells and intensifying the infection process [93]. Mangalea M.R. et al. suggest that due to compromised adaptability, changes in the bacterial genome aimed at developing simultaneous resistance to various bacteriophages from the cocktail may lead to a reduced reproduction rate, virulence and resistance to adverse factors [94].

In any case, the synergistic effect of phage cocktails makes it possible to significantly expand the range of affected bacteria and suppress their growth more effectively without causing a rapid development of phage-resistant forms. These properties of bacteriophage cocktails have been demonstrated both in our study and in many other similar studies [95,96,97].

When considering the possibility of mass use of phage preparations in the therapy of bacterial infections, special attention should be paid to the methods of their storage. These methods should ensure the preservation of high lytic properties of bacteriophages for long periods of time and be cost-effective both for storage and transportation.

A comparative investigation of three methods of storage of the developed cocktail of *E. coli* bacteriophages was performed in the course of the study: (1) storage in sterile conditions at room temperature; (2) storage in sterile conditions at 4–8 °C; (3) storage in a freeze-dried state with the addition of a stabilization medium (0.8 M sucrose solution).

The results showed that storage of the developed *E. coli* bacteriophage cocktail in sterile conditions at 4–8 °C fully preserved its original level of lytic activity. Freeze-dried cocktail reduced its lytic activity by 2 logs as compared to the freshly prepared one, but retained this level of activity throughout the entire storage period. Apparently, due to the formation of ice crystals and/or a sudden change in phase state during freeze drying, bacteriophage capsids were damaged, and this decreased the infectious titer of the cocktail [98]. The addition of sucrose stabilized the phage particles to a large extent during sublimation drying, but the destruction could not be completely avoided [99]. Storing the bacteriophage cocktail in the initial state at room temperature gradually reduced its lytic activity. This suggests a gradual degradation of phage particles takes place under such storage conditions, as was shown in earlier studies [100,101].

Thus, it was found that the initial lytic qualities of the bacteriophage cocktail are best preserved when stored in sterile conditions and at a constant temperature of 4–8 °C. However, this method requires maintaining a constant temperature, which increases the risks and economic costs. If the temperature regime is violated, the lytic activity will decrease. Freeze-drying is a more stable and cost-effective method of storage which allows the lytic activity of the phage cocktail to be preserved for a long time under standard conditions. However, it should be kept in mind that the freeze-drying process reduces the phage cocktail activity by 2 logs, even in the presence of a 0.8M sucrose solution. Hence, the initial concentration of phage particles in the freeze-dried preparation should be at least 10^7^ per 1 mL, since 10^5^ viral particles per 1 mL is considered an effective therapeutic cocktail concentration.

## 5. Conclusions

In the presented studies, six bacteriophages isolated from wastewater and capable of lysing clinical *E. coli* strains were investigated. The obtained results showed that the isolated bacteriophages had a high lytic activity and are promising candidates for the treatment of *E. coli* infections. In addition, a cocktail was developed from the isolated phages, which made it possible to increase the level of lytic activity and the host range. The possibility of long-term storage of the cocktail while maintaining its high lytic activity was also shown; this reduces the cost of producing such antibacterial preparations and makes their wider use possible.

## Figures and Tables

**Table 1:** *E. coli* strains used in the study.

*E. coli* Strains	Antibiotic Resistance	Organization Providing Bacterial Strains
444	CFZ, CAZ, LVX, CIP, AZM, PIP	JSC “Central Clinical Hospital”, Almaty
753	PEN, fluoroquinolones I-III generation, TMP	
3957	AMP, PIP, GEN, SXT	
3992	CIP, LVX	
4231	Resistance to the antibiotics used has not been identified	
4238	AMP, CXM, CTX, CAZ, FEP, GEN, CIP, LVX, SXT	
4320	Resistance to the antibiotics used has not been identified	
4325	AMP, CIP, LVX	
15597	−	ATCC

AMP—ampicillin (penicillins), AZM—azithromycin (macrolides), CAZ—ceftazidime (cephalosporins III), CFZ—cefazolin (cephalosporins I), CIP—ciprofloxacin (fluoroquinolones II), CTX—cefotaxime (cephalosporins III), CXM—cefuroxime (cephalosporins II), FEP—cefepime (cephalosporins IV), GEN—gentamicin (aminoglycosides), LVX—levofloxacin (fluoroquinolones III), PEN—penicillins, PIP—piperacillin (ureidopenicillins), SXT—trimethoprim-sulfamethoxazole, TMP—trimethoprim.

**Table 2:** Designation of isolated bacteriophages and description of the plaques.

Bacteriophage Strain	Bacteriophage Isolation Place	Host Bacterium Strain	Plaques
vB_EcoM_SCS4	Sewage collector of Shymkent, Kazakhstan	*E. coli* 753	Limpid plaque ≈4 mm in diameter with a slight halo up to 8 mm
vB_EcoS_SCS44		*E. coli* 444	Limpid plaque with a diameter of ≈2 mm without halo
vB_EcoM_SCS57		*E. coli* 3957	Limpid plaque with a diameter of ≈1.5 mm without halo
vB_EcoS_SCS92		*E. coli* 3992	Limpid plaque with a diameter of ≈2 mm without halo
vB_EcoD_SCS31		*E. coli* 4231	Limpid plaque with a diameter of ≈4 mm with a pronounced halo up to 6 mm
vB_EcoP_FFK3	Sewage disposal fields of Konaev, Kazakhstan	*E. coli* 753	A limpid plaque ≈4 mm in diameter with a slight halo up to 7 mm.

**Table 3:** Key feature of the genomes.

Phages	Length (bp)	No. of ORFs	No. of tRNAs	GC Content (%)
vB_EcoM_SCS57	168,771	272	2	37.6
vB_EcoM_SCS4	166,783	269	0	35.4
vB_EcoS_SCS92	44,240	72	0	51.0
vB_EcoS_SCS44	44,829	78	0	50.6
vB_EcoS_SCS31	46,054	78	1	43.5
vB_EcoP_FFK3	49,300	48	0	49.8

**Table 4:** The level of lytic activity of the *E. coli* bacteriophages.

*E. coli* Bacteriophage Strain	MOI							
	1	10^−1^	10^−2^	10^−3^	10^−4^	10^−5^	10^−6^	10^−7^
vB_EcoM_SCS4	++ *	++	++	++	++	++	++	++
vB_EcoS_SCS44	++	++	++	++	+	+	+	+
vB_EcoM_SCS57	++	++	++	++	++	++	+	+
vB_EcoS_SCS92	++	++	++	++	++	++	++	+
vB_EcoS_SCS31	++	++	++	++	++	++	++	++
v_BEcoP_FFK3	++	++	++	++	++	++	++	+

*++—suppression of the growth of the bacterial test culture by 90–100%; +—suppression of the growth of the bacterial test culture by 50–90%. 100% inhibition of bacterial growth—optical density value of samples without the addition of bacterial suspension (negative control), 0% inhibition of growth of bacteria—optical density value of samples without the addition of phage lysates (positive control).

**Table 5:** Host range of *E. coli* bacteriophages.

Bacteriophage Strain	Clinical Strains of *E. coli*								
	444	753	3957	3992	4231	4238	4320	4325	15597
vB_EcoM_SCS4	− *	+++	−	−	+++	+++	+++	+++	+++
vB_EcoS_SCS44	+++	−	++	−	−	−	++	−	−
vB_EcoM_SCS57	+++	+	+++	−	++	−	+++	−	+++
vB_EcoS_SCS92	++	−	+++	+++	−	−	−	−	−
vB_EcoS_SCS31	++	−	−	−	+++	−	−	−	−
v_BEcoP_FFK3	−	+++	−	−	+++	−	−	+++	−

*—+++—inhibition of bacterial culture growth by 90–100%; ++—inhibition of bacterial culture growth by 90–50%; +—inhibition of bacterial culture growth by 20–50%; −—lack of inhibition of bacterial culture growth. 100% inhibition of bacterial growth—optical density value of samples without the addition of bacterial suspension (negative control), 0% inhibition of growth of bacteria—optical density value of samples without the addition of phage lysates (positive control).

## Data Availability

The genome sequences were deposited in GenBank under the accession numbers OM960734, ON001686, ON081052, ON506924, ON548432, ON548433. The raw data supporting the conclusions of this article will be made available by the authors, without undue reservation.

## Supplementary Materials

The following supporting information can be downloaded at: https://www.mdpi.com/article/10.3390/v14112381/s1, Figure S1: Genome map of the vB_EcoM_SCS57; Figure S2: Genome map of the vB_EcoM_SCS4; Figure S3: Genome map of the vB_EcoS_SCS92; Figure S4: Genome map of the vB_EcoS_SCS44; Figure S5: Genome map of the vB_EcoS_SCS31; Figure S6: Genome map of the vB_EcoP_FFK3; Figure S7: BRIG representation reflecting the complete genomic comparison of other phages against vB_EcoM_SCS57 phage; Figure S8: BRIG representation reflecting the complete genomic comparison of other phages against vB_EcoM_SCS4 phage; Figure S9: BRIG representation reflecting the complete genomic comparison of other phages against vB_EcoS_SCS92 phage; Figure S10: BRIG representation reflecting the complete genomic comparison of other phages against vB_EcoS_SCS44 phage; Figure S11: BRIG representation reflecting the complete genomic comparison of other phages against vB_EcoS_SCS31 phage; Figure S12: BRIG representation reflecting the complete genomic comparison of other phages against vB_EcoP_FFK3 phage.
