# Supplementary figures and images for: Isolation and Characterization of Lytic Bacteriophages Active against Clinical Strains of E. coli and Development of a Phage Antimicrobial Cocktail
Source: Viruses. 2022 Oct 28;14(11):2381. doi: 10.3390/v14112381 (PMC9697832; doi:10.3390/v14112381)

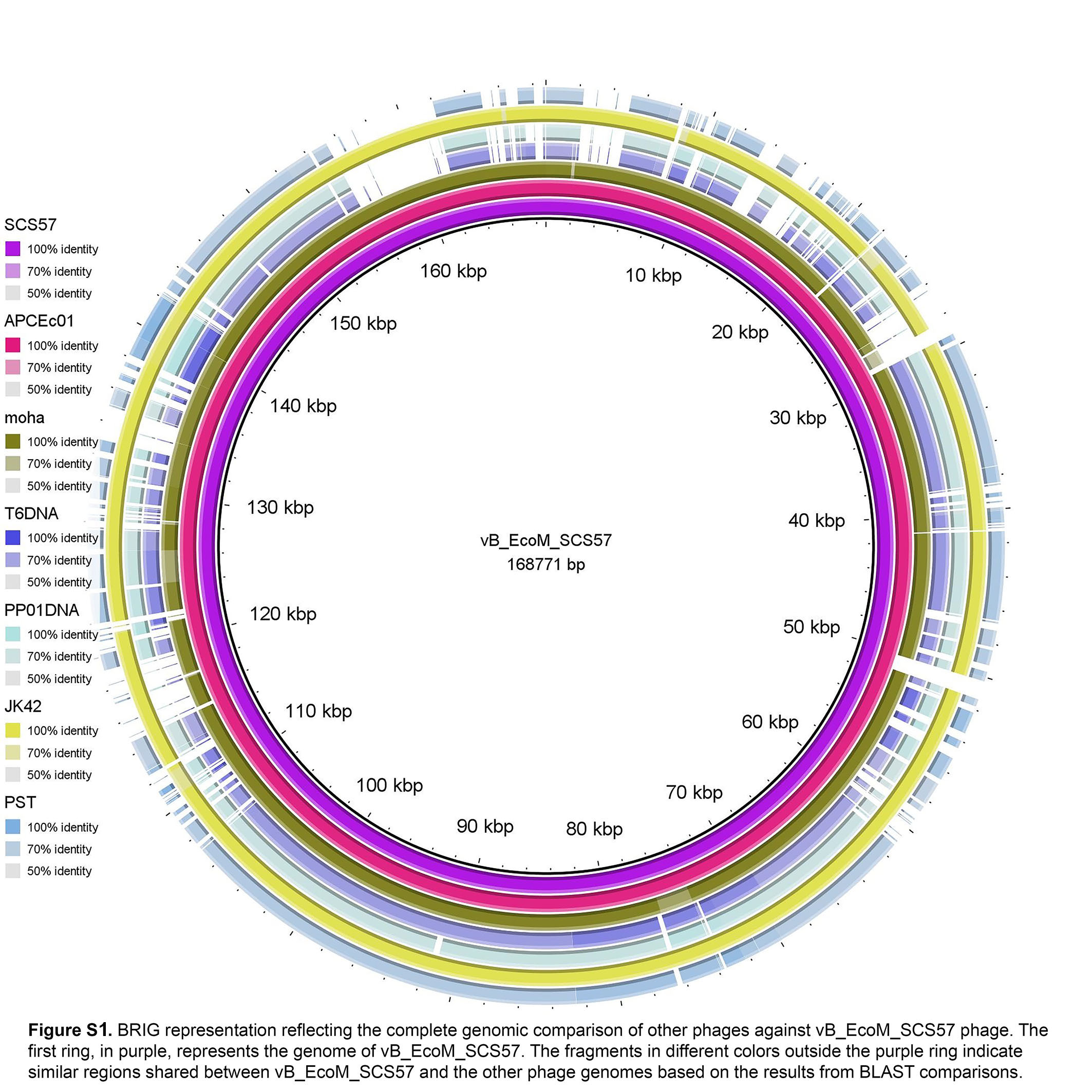

Supplement: Supplementary file 1 [file viruses-14-02381-s001.zip › Supplementary Figure S1.jpg]

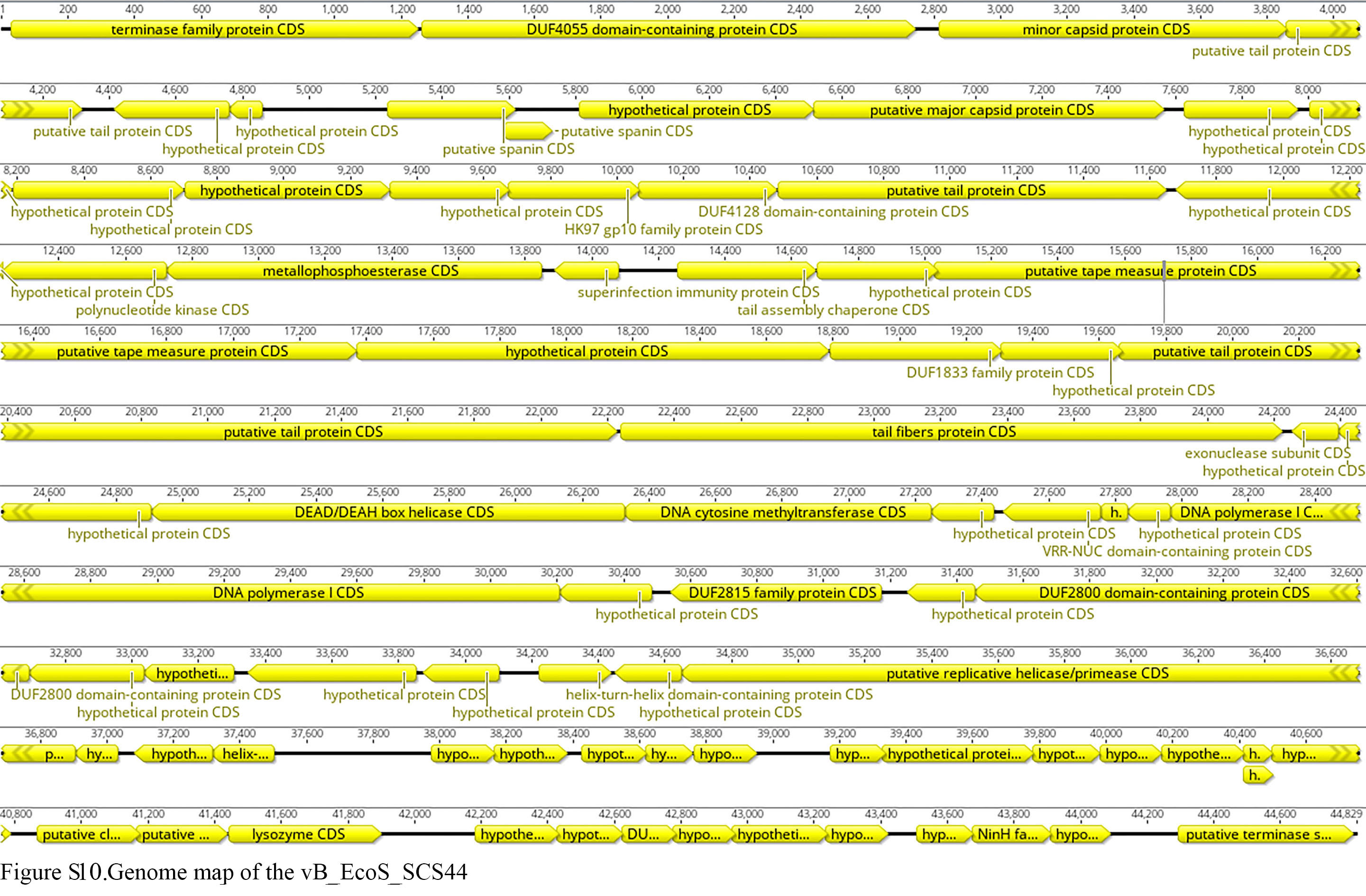

Supplement: Supplementary file 1 [file viruses-14-02381-s001.zip › Supplementary Figure S10.jpg]

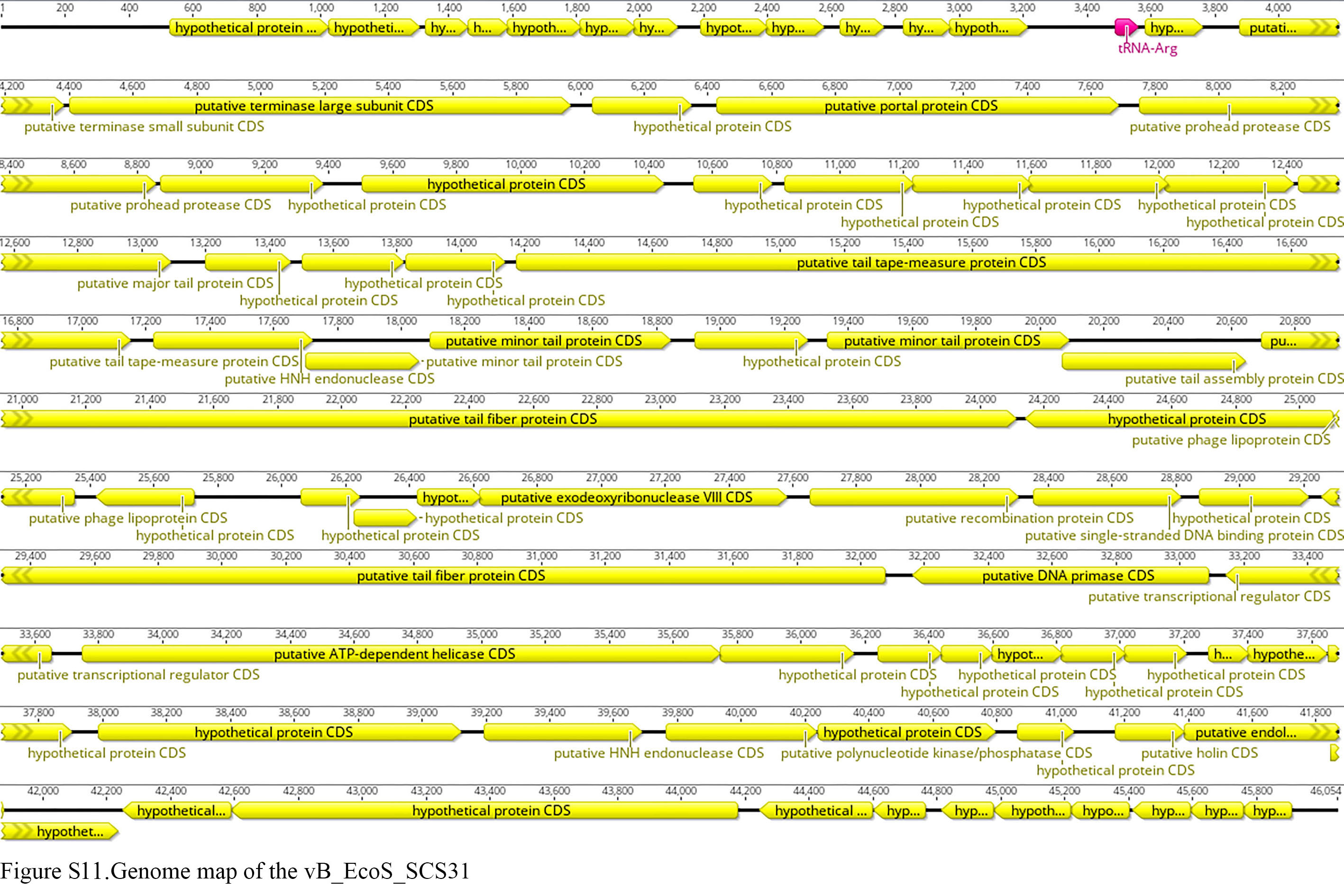

Supplement: Supplementary file 1 [file viruses-14-02381-s001.zip › Supplementary Figure S11.jpg]

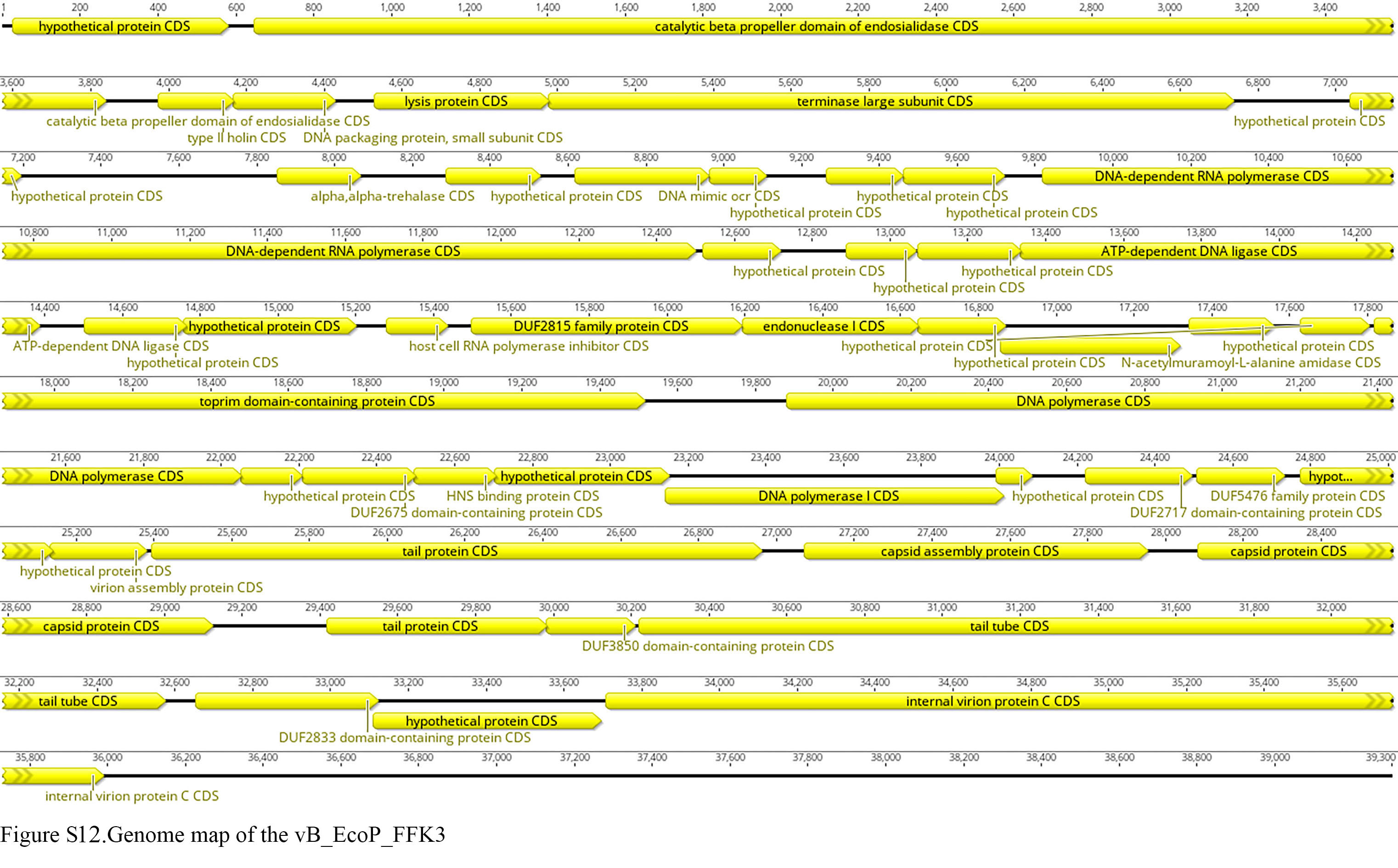

Supplement: Supplementary file 1 [file viruses-14-02381-s001.zip › Supplementary Figure S12.jpg]

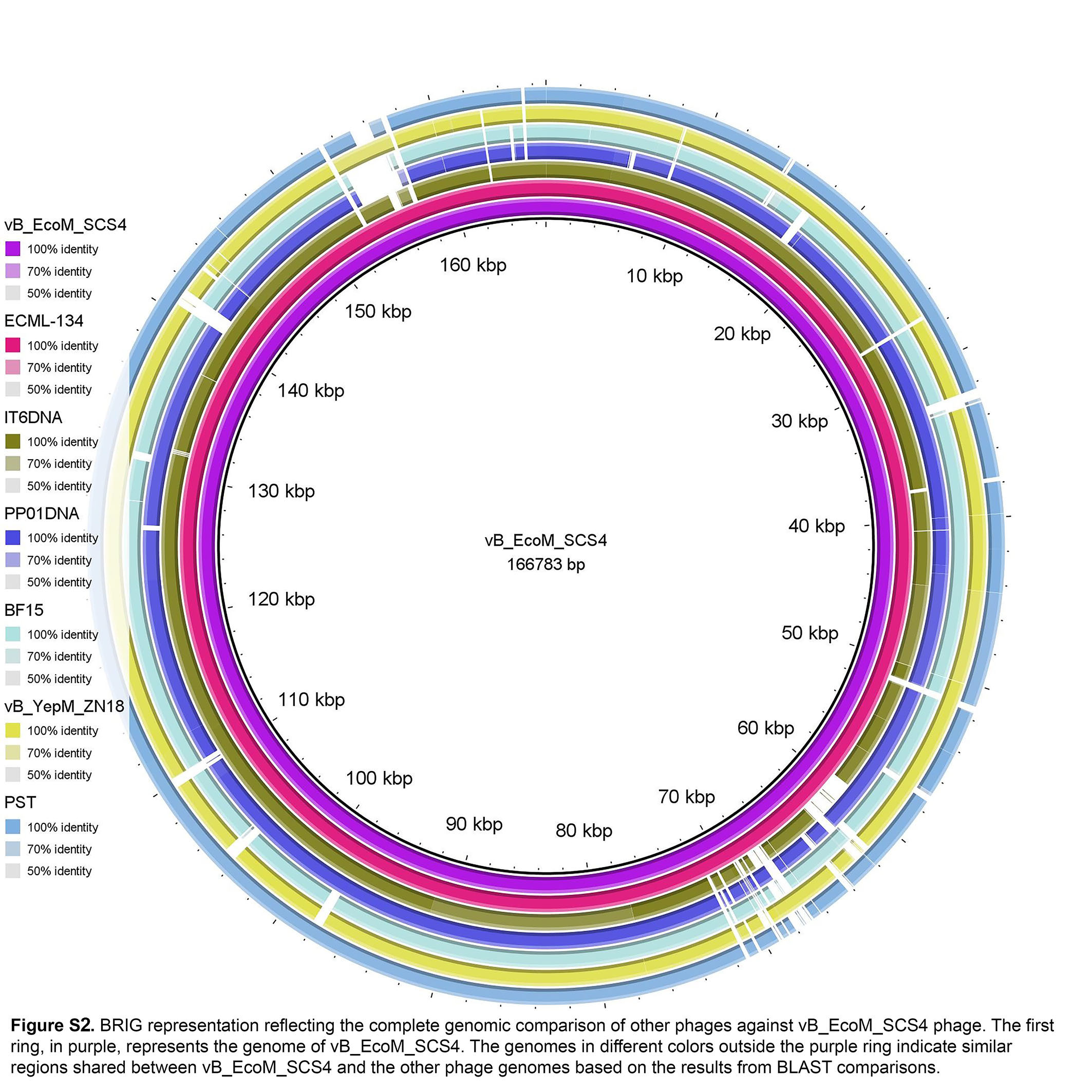

Supplement: Supplementary file 1 [file viruses-14-02381-s001.zip › Supplementary Figure S2.jpg]

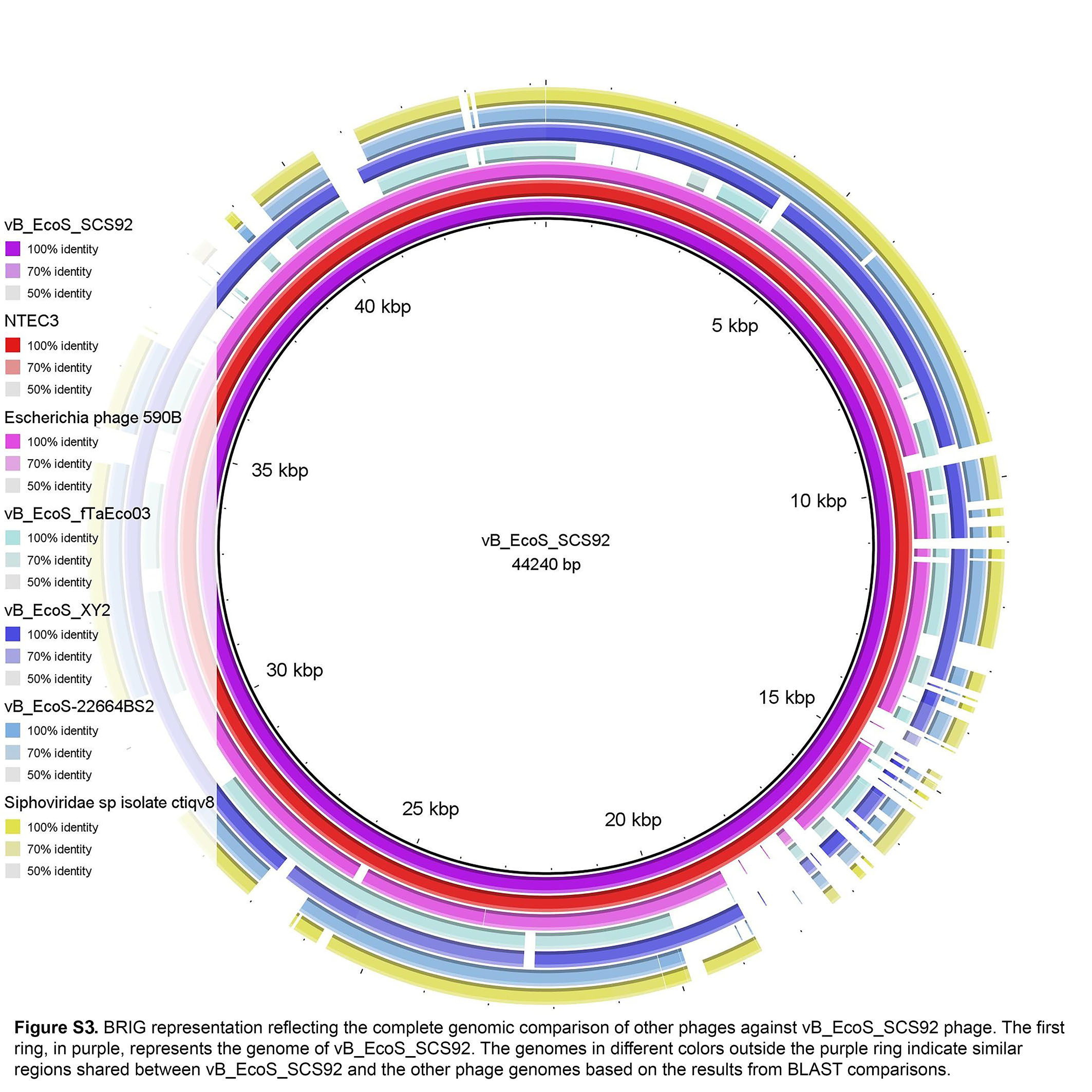

Supplement: Supplementary file 1 [file viruses-14-02381-s001.zip › Supplementary Figure S3.jpg]

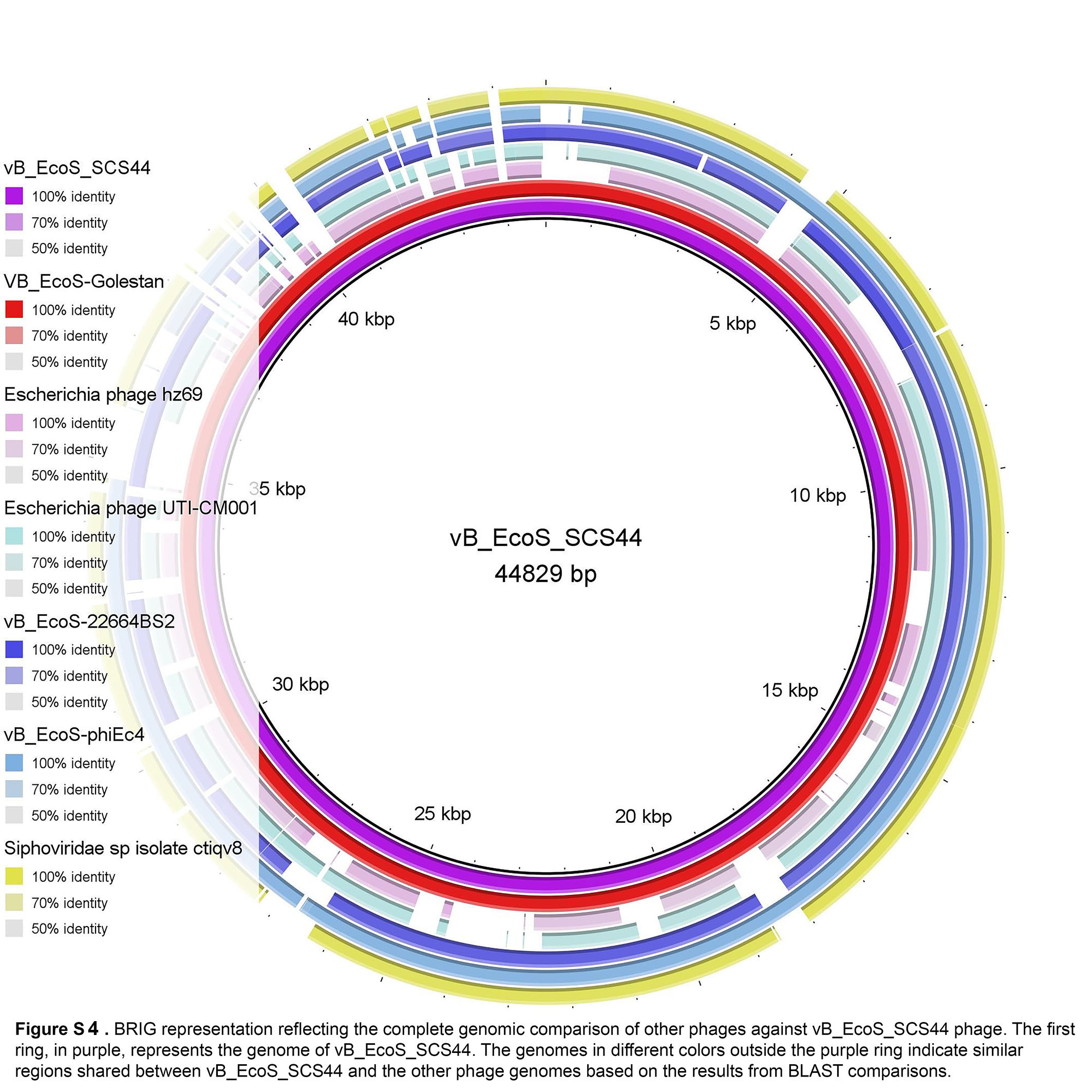

Supplement: Supplementary file 1 [file viruses-14-02381-s001.zip › Supplementary Figure S4.jpg]

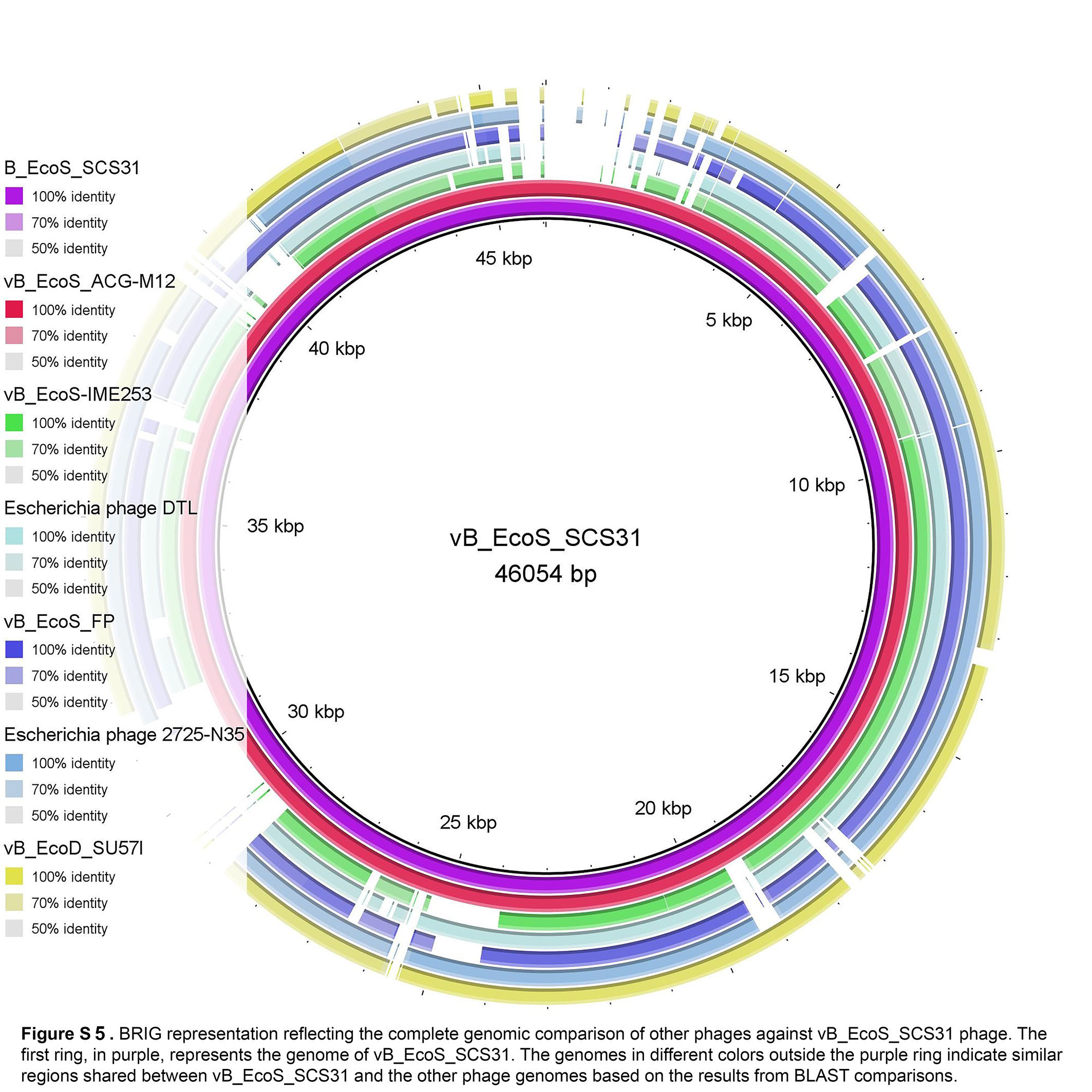

Supplement: Supplementary file 1 [file viruses-14-02381-s001.zip › Supplementary Figure S5.jpg]

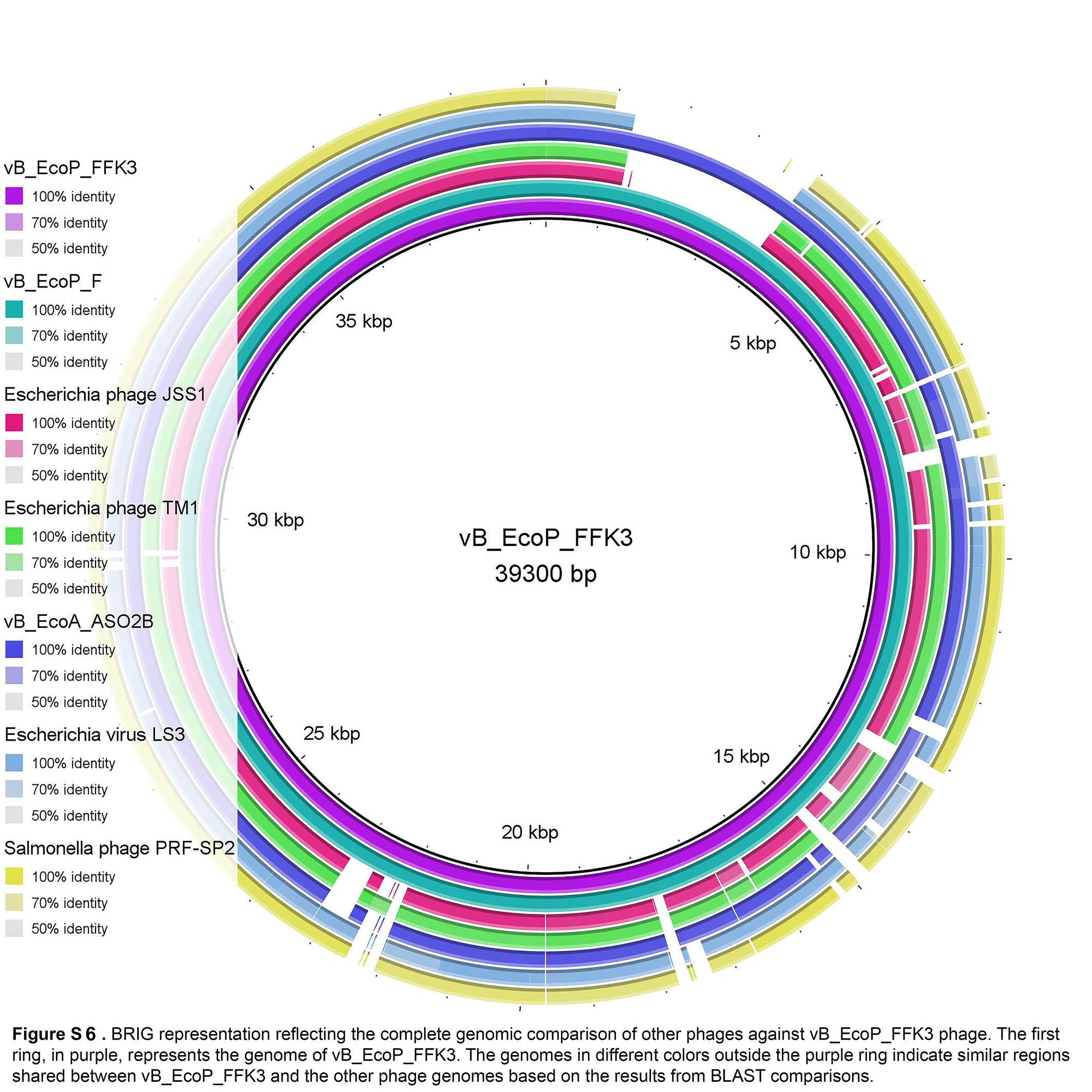

Supplement: Supplementary file 1 [file viruses-14-02381-s001.zip › Supplementary Figure S6.jpg]

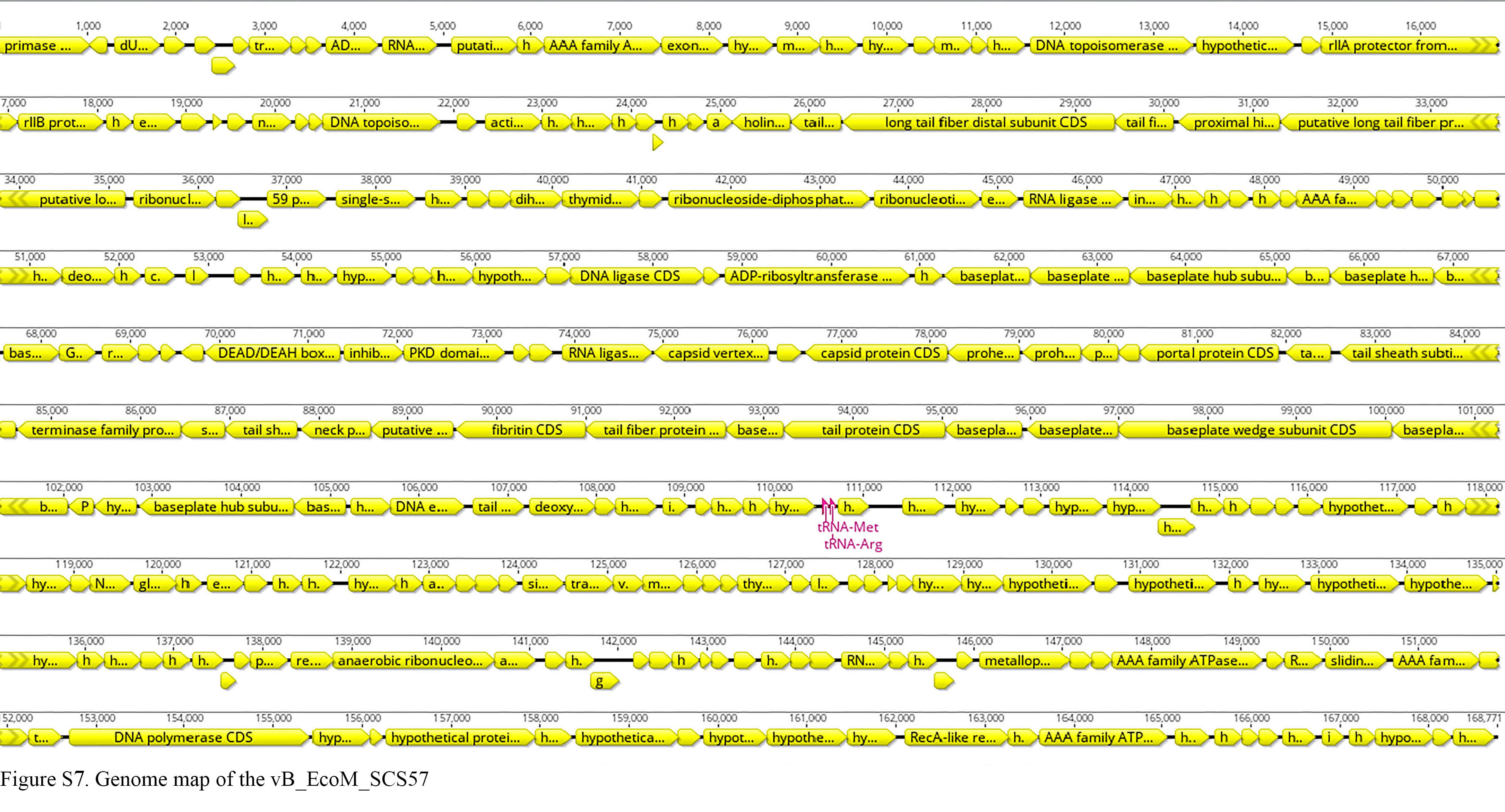

Supplement: Supplementary file 1 [file viruses-14-02381-s001.zip › Supplementary Figure S7.jpg]

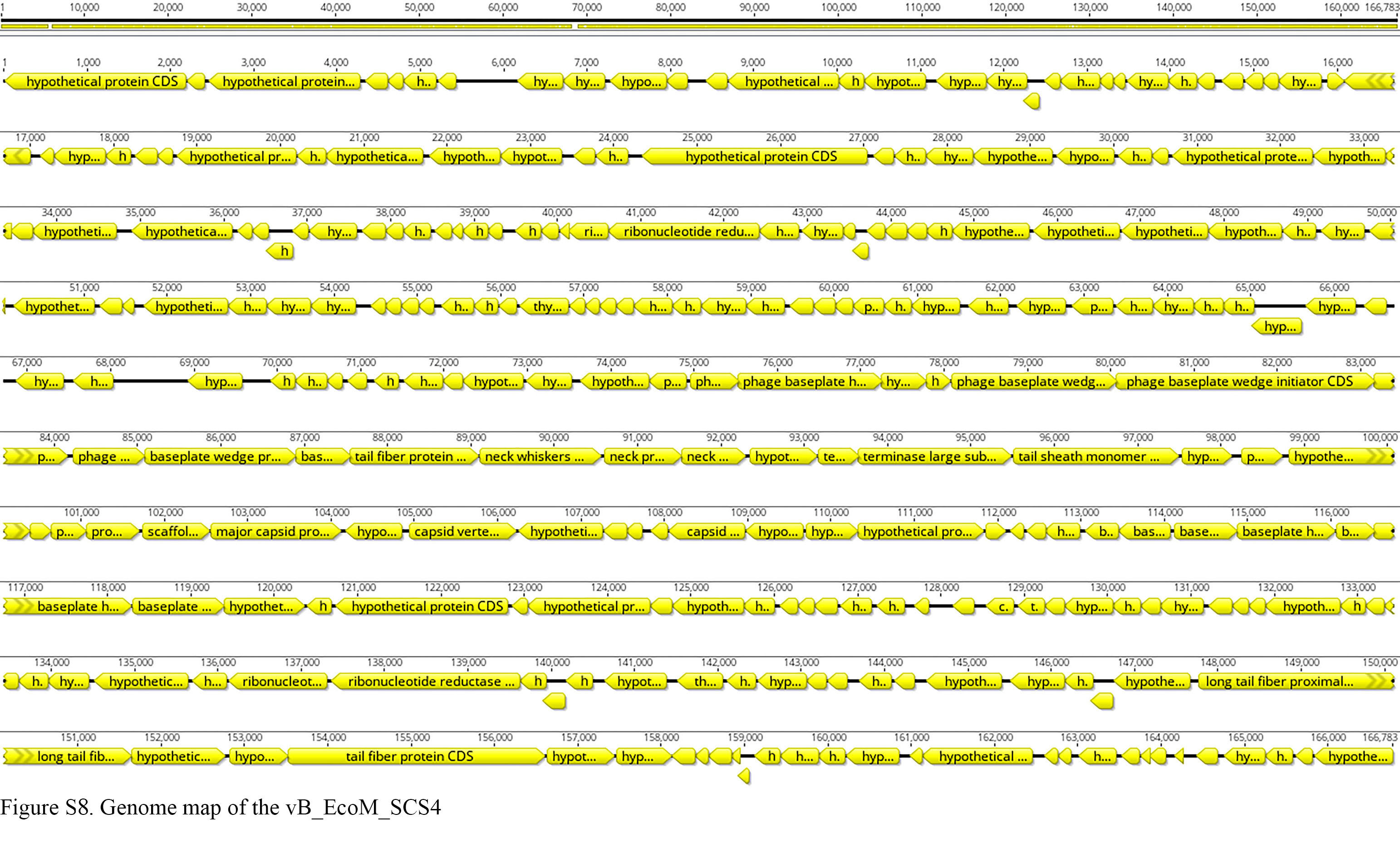

Supplement: Supplementary file 1 [file viruses-14-02381-s001.zip › Supplementary Figure S8.jpg]

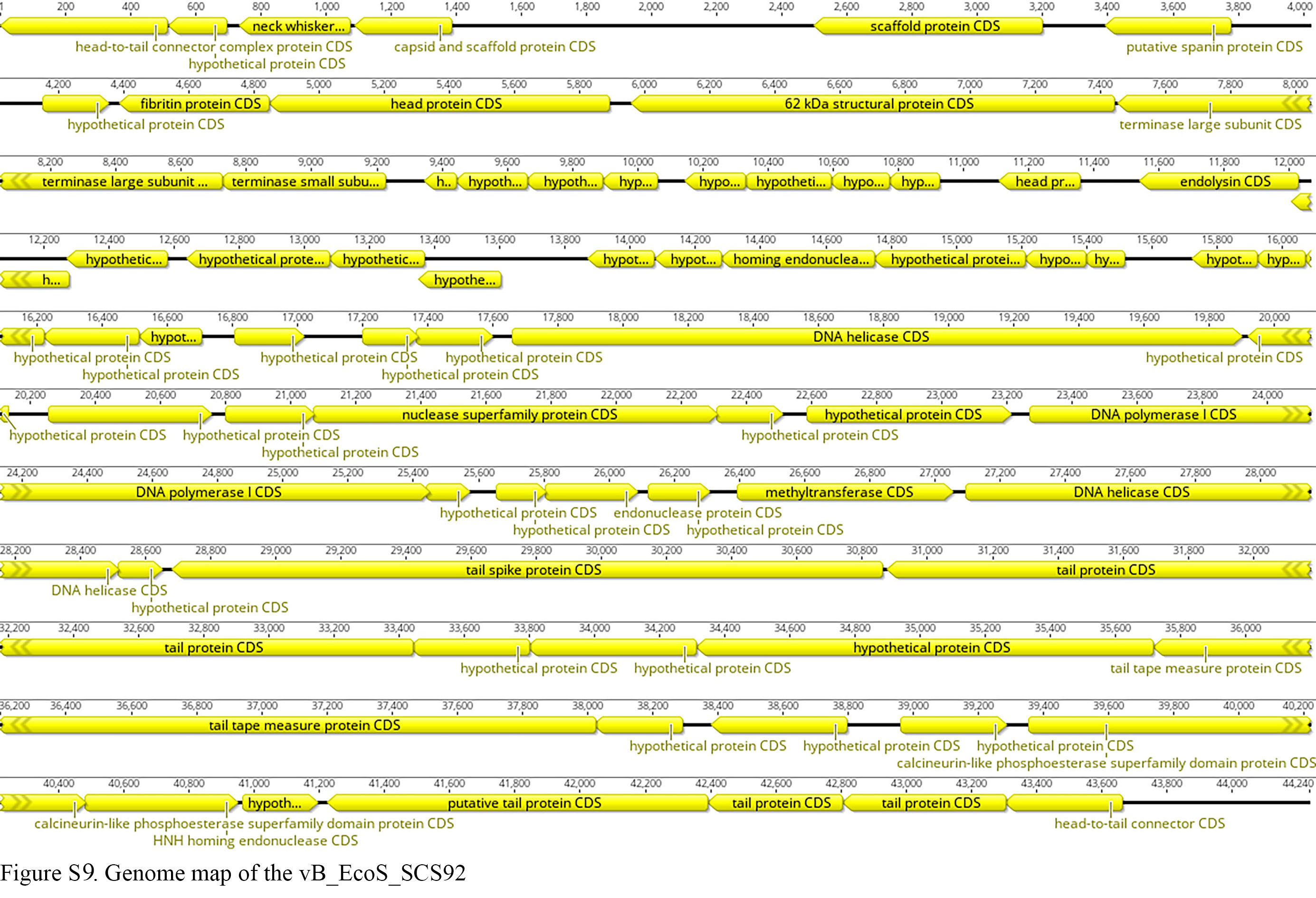

Supplement: Supplementary file 1 [file viruses-14-02381-s001.zip › Supplementary Figure S9.jpg]
